# Cathode Electrolyte Interphase Engineering by Quaternized Chitosan for Stabilized Li–SPAN Batteries

**DOI:** 10.1002/advs.202515551

**Published:** 2025-11-21

**Authors:** Runhe He, Hao Liu, Qing Gao, Dong Cai, Kuikui Xiao, Yinhang Zhang, Xinmin Zhang, Xianyang Cheng, Yuhui Wang, Lixing Kang, Huagui Nie, Yatao Liu, Zhi Yang

**Affiliations:** ^1^ Key Laboratory of Carbon Materials of Zhejiang Province Wenzhou University Wenzhou 325035 China; ^2^ Key Laboratory of Multifunctional Nanomaterials and Smart Systems Division of Advanced Materials Suzhou Institute of Nano‐Tech and Nano‐Bionics Chinese Academy of Sciences Suzhou 215123 China; ^3^ College of Materials Science and Engineering University of Jinan Jinan 250022 China; ^4^ State Key Laboratory of Organic‐Inorganic Composites College of Chemical Engineering Beijing University of Chemical Technology Beijing 100029 China

**Keywords:** cathode electrolyte interphase, interface engineering, lithium−sulfur battery, quaternized chitosan, sulfurized polyacrylonitrile

## Abstract

Sulfurized polyacrylonitrile (SPAN) represents a highly promising cathode material for lithium–sulfur (Li–S) batteries, leveraging a solid‐solid sulfur conversion mechanism. However, persistent interfacial side reactions and sluggish redox kinetics in SPAN cathodes compromise the electrochemical performance. Here, quaternized chitosan (QCS) is employed as a functional agent to stabilize the SPAN cathode electrolyte interphase (CEI). The positively charged quaternary ammonium groups selectively adsorb PF_6_^–^ anions, modifying the Helmholtz layer structure and facilitating the formation of an anion‐derived CEI enriched with LiF. Consequently, the SPAN@QCS‐1.0% cathode delivers a high discharge capacity of 1499 mAh g^−1^ at 0.2 C, an outstanding rate capability of 902 mAh g^−1^ at 10 C, and a prolonged cycle life exceeding 1500 cycles at 1 C. Under practical conditions of high sulfur loading (12.0 mg cm^−2^) and lean electrolyte (E/S ratio = 5 µL mg^−1^), the SPAN cell achieves a high areal capacity of 17.1 mAh cm^−2^, surpassing that of conventional lithium–ion batteries (≈4 mAh cm^−2^) by more than fourfold. Furthermore, a 0.9 Ah pouch‐cell prototype demonstrates stable cycling for over 30 cycles. The interface strategy provides a facile and effective approach to developing high‐performance SPAN‐based Li–S batteries.

## Graphical abstract

In this work, quaternized chitosan (QCS) is reported as an additive optimizing the sulfurized polyacrylonitrile (SPAN) cathode/electrolyte interphase (CEI). Its high positive charge density adsorbs PF_6_^−^ anions, enriching the Helmholtz layer with aggregates. This promotes rapid anion desolvation, forming an anion‐derived CEI enriched in LiF. The resulting robust, ion‐conductive CEI significantly enhances Li‐SPAN cell electrochemical performance.

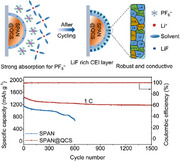

## 1 Introduction

Conventional lithium–ion (Li–ion) batteries, based on insertion/extraction chemistry, are approaching their practical energy density limits (≈300 Wh kg^−1^) and struggle to meet the rising demands of energy‐storage markets, including drones, portable electronics, and long‐distance transportation.^[^ ^1^, ^2^, ^3^ ^]^ Lithium–sulfur (Li–S) batteries have emerged as a promising alternative, offering exceptional theoretical energy density (≈2600 Wh kg^−1^) coupled with the cost‐effectiveness of sulfur (≈$0.25 kg^−1^).^[^ ^4^, ^5^, ^6^ ^]^ The chemistry leverages the high theoretical capacity of sulfur (S_8_, 1675 mAh g^−1^) through multistep conversion between insulating S_8_ and lithium sulfide (Li_2_S). However, this process involves sluggish kinetics, significant volume changes (up to ≈80%), and the formation of soluble polysulfide intermediates (Li_2_S_x_, x ≥ 4).^[^ ^7^, ^8^ ^]^ Polysulfide dissolution leads to active material loss and shuttling, causing severe capacity degradation and poor cycling efficiency, particularly under practical conditions.^[^ ^9^, ^10^, ^11^, ^12^ ^]^

A promising strategy for these challenges is to employ solid‐state sulfur conversion chemistry by covalently conjugating sulfur moieties with conductive polymer‐derived carbon architectures.^[^ ^13^, ^14^, ^15^ ^]^ Exemplified by sulfurized polyacrylonitrile (SPAN) composites—first synthesized by Wang et al. in 2002,^[^ ^16^ ^]^ this design eliminates soluble polysulfide intermediates, significantly enhancing cyclability and Coulombic efficiency. While the precise structure and electrochemical mechanism of SPAN remain debated, consensus indicates sulfur is covalently bonded to the PAN‐derived carbon matrix, with redox processes involving only short‐chain lithium polysulfides (Li_2_S_x_, x ≤ 4).^[^ ^17^, ^18^, ^19^ ^]^ This solid‐solid conversion mechanism enhances cathode stability and electrochemical performance.

Despite these advances, SPAN composites face persistent limitations in long‐term operational durability, notably accelerated capacity fade during extended cycling. Huang et al. observed rapid degradation in pristine SPAN cathodes beyond 150 cycles,^[^ ^20^ ^]^ and studies by Wang's group^[^ ^21^ ^]^ noted instability in standard carbonate electrolytes (1 m LiPF_6_/EC:DMC, 1:1 v/v). Recent studies underscore that the cathode‐electrolyte interphase (CEI) is imperative for high performance, given that well‐engineered CEI architectures facilitate the mitigation of interfacial side reactions and promote superior electrode stability.^[^ ^22^, ^23^, ^24^ ^]^ Emerging electrolyte engineering strategies, including high‐concentration electrolytes (HCEs) and localized high‐concentration electrolytes (LHCEs), have demonstrated effectiveness in modulating solvation structures to form robust cathode–CEI layers, thereby extending the cycle life of Li–SPAN cells.^[^ ^25^, ^26^, ^27^ ^]^ In HCE systems, the elevated salt‐to‐solvent ratio reduces the population of free solvent molecules and increases the participation of anions in Li^+^ solvation. Consequently, the resulting CEI is predominantly derived from the extensive anion decomposition. For instance, an ether‐based HCE incorporating LiTFSI and LiNO_3_ as co‐salts has been shown to facilitate the formation of a CEI composed of LiF and LiNO_2_ on the SPAN surface.^[^ ^28^ ^]^ This interphase effectively suppresses the formation of soluble polysulfides and enables stable cycling of Li–SPAN batteries. However, concentrated electrolytes are often hampered by high viscosity and cost. The introduction of fluorinated ether diluents can decrease the electrolyte viscosity and reduce lithium salt consumption. For example, in an LHCE system incorporating the ionic liquid Py_13_TFSI, a coordinated‐anion‐enriched solvation structure forms, leading to a LiF–Li*_x_* N*_y_* O_2_‐rich CEI.^[^ ^29^ ^]^ This LiF‐dominated interphase accommodates large volume changes in SPAN cathodes, significantly improving cycling stability even under high areal capacity conditions. Nevertheless, the high cost of fluorinated diluents—often exceeding that of lithium salts—and the complex optimization of solvent/salt/diluent ratios in LHCE systems remain challenging. A simple and cost‐effective approach to tailor the SPAN electrode–electrolyte interphase is still essential for the practical implementation of Li–S batteries.

Surface modification offers another viable route to stabilize the cathode–electrolyte interface.^[^ ^30^ ^]^ For example, coating CoS_2_ onto SPAN–CNT fibers has enabled the fabrication of a self‐supporting sulfur cathode with ultrahigh areal capacity.^[^ ^31^ ^]^ Similarly, depositing an inorganic carbon layer on SPAN mitigates interfacial side reactions and improves cycling stability.^[^ ^32^ ^]^ Other reported coating includes graphene,^[^ ^33^ ^]^ polydopamine (PDA),^[^ ^34^ ^]^ and cross‐linked PDA/PAA/PVA.^[^ ^35^ ^]^ However, such coating layers are typically electrochemically inert and function primarily by physically isolating active materials from the electrolyte. A more advanced strategy involves designing functional interlayers that selectively adsorb specific molecules or ions within the Helmholtz layer to guide preferential decomposition. Quaternized chitosan (QCS)—a cationic chitosan derivative containing quaternary ammonium groups—exhibits high charge density, excellent water solubility, and selective adsorption, film‐forming, and permeability properties. The rationale for using QCS lies in its high‐density positive charges, which enable selective adsorption of PF_6_^–^ anions from the electrolyte. Unlike bulk electrolyte engineering, this approach enables precise tuning of the interfacial electrolyte environment without compromising bulk ionic conductivity or increasing viscosity.

**Scheme 1:**
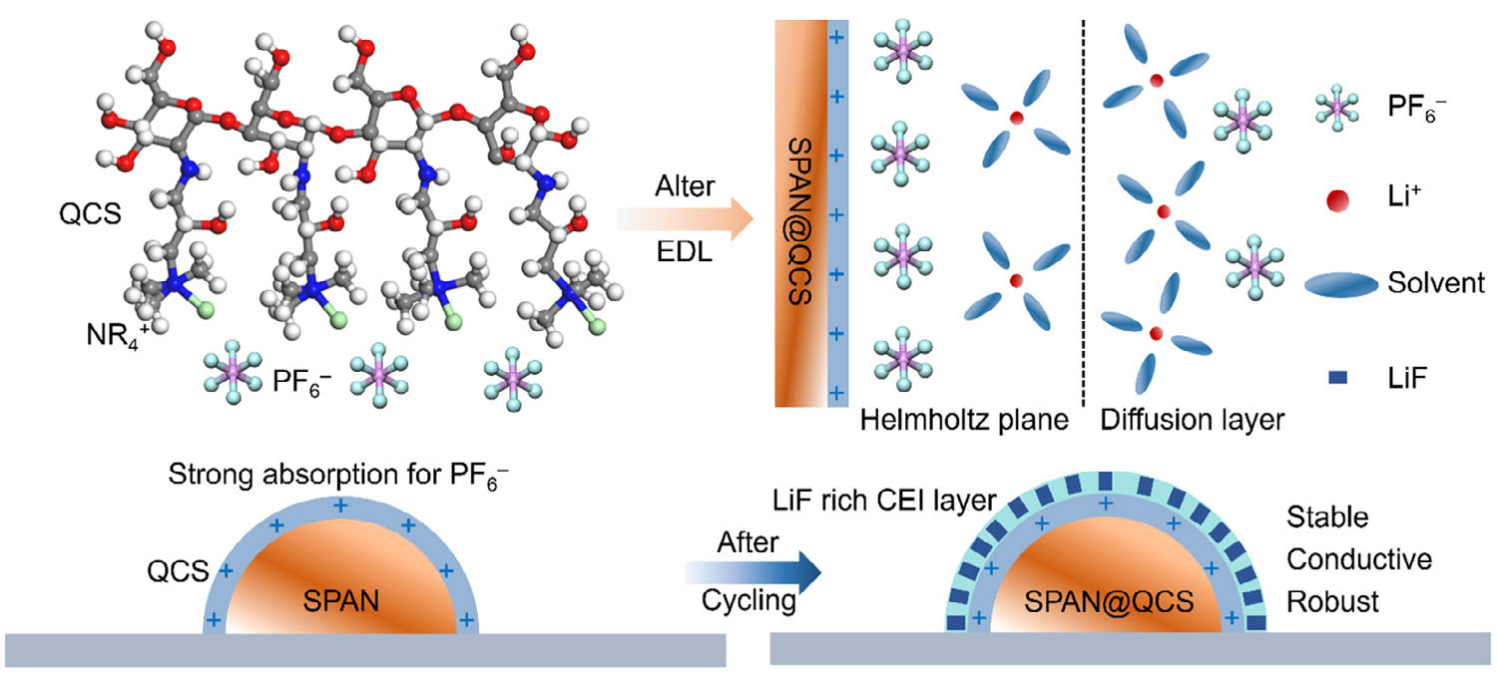
Schematic illustration of the CEI engineering by QCS for SPAN cathode.

Herein, we applied QCS as a functional agent to modify the CEI on SPAN cathodes. Its high‐density positive charges selectively adsorb PF_6_^–^ anions, enriching the Helmholtz layer with PF_6_^–^ aggregates. This drives rapid anion de‐solvation and the formation of an anion‐derived CEI enriched with LiF, enhancing mechanical stability, interfacial robustness, and Li^+^ conductivity (**Scheme** **1**). Consequently, the SPAN@QCS‐1.0% cathodes deliver a prolonged cycle life exceeding 1500 cycles at 1 C and a high‐rate capacity of 902 mAh g^−1^ at 10 C. Under demanding conditions of high sulfur loading (12.0 mg cm^−2^) and low electrolyte/sulfur (E/S) ratio (5 µL mg^−1^), it achieves a high areal capacity of 17.1 mAh cm^−2^, surpassing that of conventional lithium–ion batteries (≈4 mAh cm^−2^) by more than fourfold. More importantly, the Ah‐level pouch cells demonstrate stable cycling for over 30 cycles, demonstrating good practicality for Li–S batteries.

## 2 Results and Discussion

**Figure 1:**
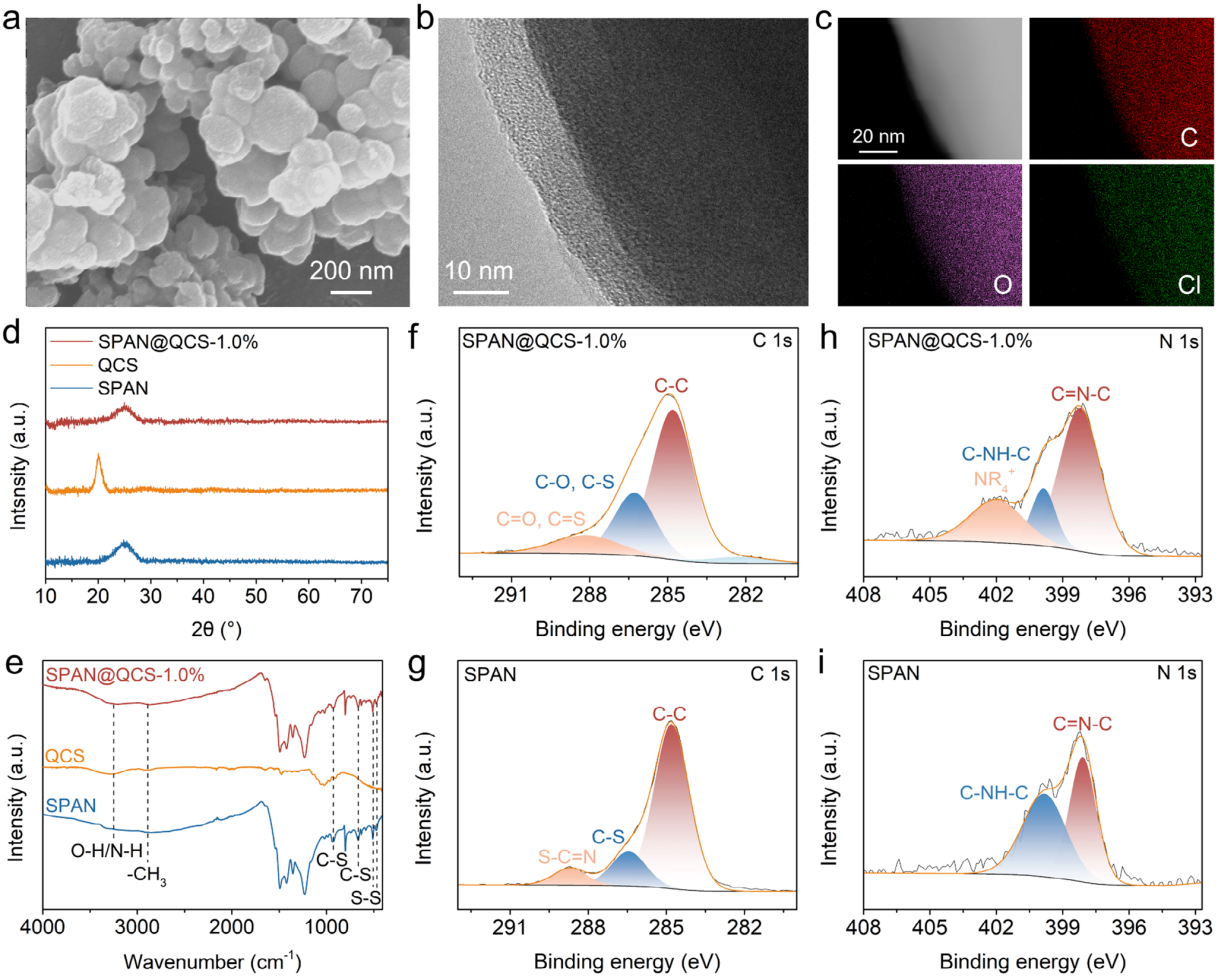
Materials characterization. a) SEM and b) HR‐TEM images of SPAN@QCS‐1.0% cathode. c) dark‐field TEM image, and corresponding elemental mappings of SPAN@QCS‐1.0%. d) XRD and e) FTIR of SPAN, QCS, and SPAN@QCS‐1.0%. f,g) C 1s XPS spectra of f) SPAN@QCS‐1.0% and g) SPAN. h,i) N 1s XPS spectra of h) SPAN@QCS‐1.0% and i) SPAN.

SPAN was synthesized via thermal pyrolysis of PAN in the presence of elemental sulfur. Upon heating, sulfur can dehydrogenate PAN to generate conductive frameworks by forming stable heterocyclic rings, which is further dehydrogenated and substituted by sulfur to form cross‐linking C–S*_x_*–C chains or ring structures. The scanning electron microscopy (SEM) images show that SPAN exhibits spheroid particles with an average size ≈200 nm (**Figure** **1**a). Raman spectra reveal the *sp* ^3^ C─C bonds (D band, 1320 cm^−1^) and *sp* ^2^ C═C bonds (G band, 1530 cm^−1^) in SPAN backbones, as well as the C─S bond (179 and 367 cm^−1^) and S─S bond (461 and 930 cm^−1^) (Figure S1, Supporting Information).^[^ ^36^, ^37^ ^]^ The X‐ray photoelectron spectroscopy (XPS) analysis (Figure S2, Supporting Information) confirms the C─S bond (161.3 and 162.5 eV) and S─S bond (163.3 and 164.5 eV) of amorphous sulfur phase or short‐chain organosulfide in the composite.^[^ ^38^ ^]^ The elemental analysis shows that the sulfur content in SPAN is ≈47% (Table S1, Supporting Information).

QCS (1.0 wt.%) was added to the cathode slurry as a functional additive. QCS can be well dissolved in the slurry and coats uniformly on the SPAN particles owing to its excellent film‐forming capability. The SEM images of the cathode show a uniform and continuous structure (Figure S3, Supporting Information). There are no visible aggregates of binder or QCS, and the SPAN particles appear to be well‐integrated into a cohesive structure, which is indicative of a uniform binder and QCS distribution. High‐resolution transmission electron microscopy (HR‐TEM) reveals a continuous, homogeneous coating layer ≈10 nm thick (Figure 1b). Elemental mapping indicates that C, O, and Cl are uniformly distributed on the surface of SPAN (Figure 1c), further demonstrating the uniform encapsulation of QCS. X‐ray diffraction (XRD) patterns of SPAN show one broad diffraction peak at ≈25° (Figure 1d), attributed to the 200 plane of graphite carbon.^[^ ^39^ ^]^ Due to the very low content of QCS, the typical peak at 20° relating to QCS is hardly observed for SPAN@QCS‐1.0% samples. Fourier transform infrared spectroscopy (FTIR) of pristine SPAN (Figure 1e) shows peaks at 471 and 513 cm^−1^ (S−S symmetric stretching) and 665 and 926 cm^−1^ (C─S bonds).^[^ ^40^, ^41^ ^]^ For SPAN@QCS‐1.0%, new peaks emerge at 2895 cm^−1^ (─CH_3_) and 3250 cm^−1^ (O─H/N─H vibrations).^[^ ^42^, ^43^ ^]^ Figure 1f,g shows the C 1s XPS spectra for the pristine SPAN and SPAN@QCS‐1.0%, respectively. Compared to the bare SPAN, the C 1s peak at 285.9 eV (C─O bonds) for the SPAN@QCS‐1.0% samples are significantly enhanced, indicating that QCS is successfully coated on the SPAN surface.^[^ ^44^ ^]^ In addition, the appearance of a new strong N 1s peak at 402.0 eV (NR_4_^+^) provides another evidence for the SPAN decorated with the QCS layer (Figure 1h,i).^[^ ^45^ ^]^

**Figure 2:**
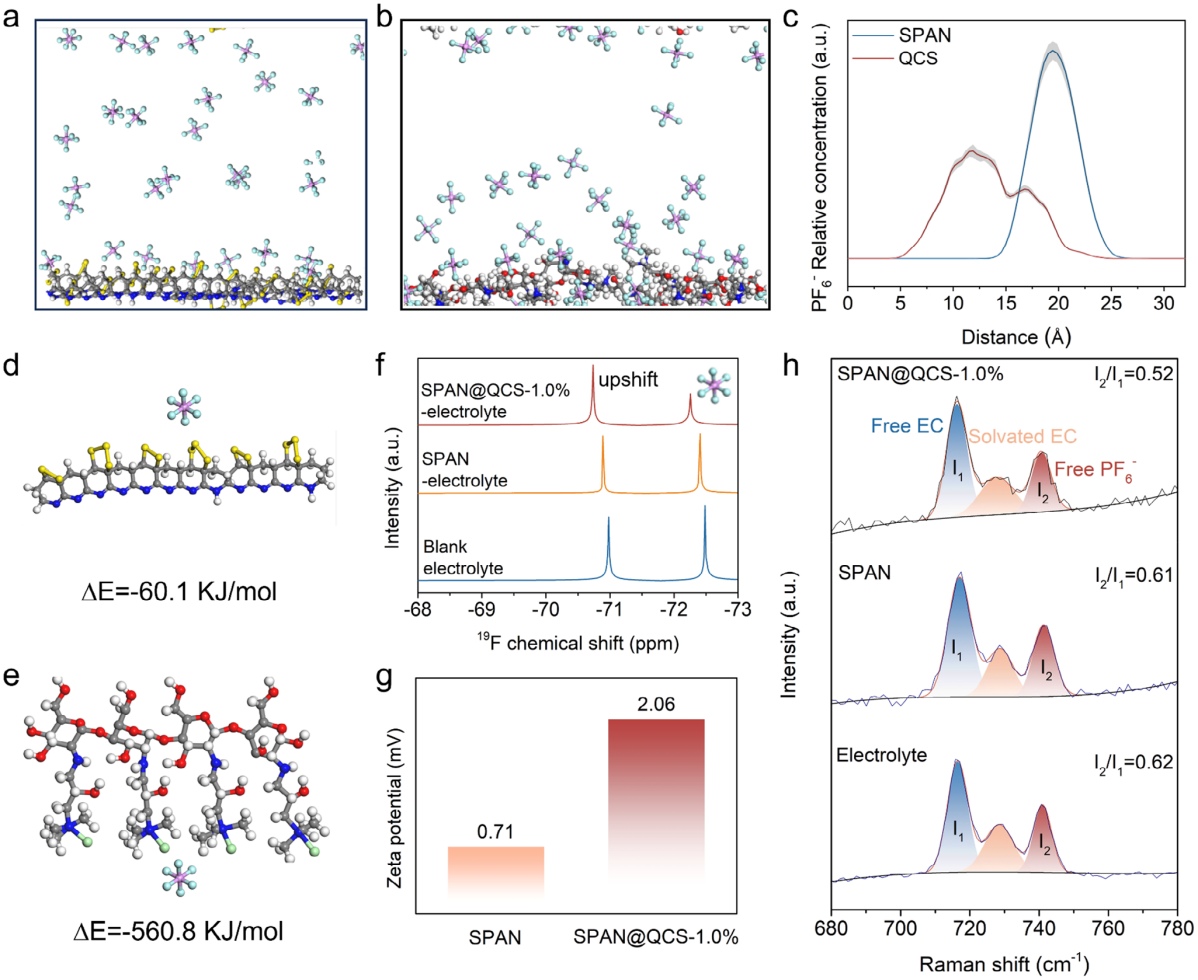
MD simulations and adsorption analysis. a,b) MD simulations. c) PF_6_^−^ adsorption profiles on SPAN and QCS surfaces. d,e) Adsorption configuration and energies of PF_6_^−^ with SPAN and QCS. f) ^19^F NMR spectra of the electrolyte before and after soaking SPAN and SPAN@QCS‐1.0%. g) Zeta potentials of SPAN and SPAN@QCS‐1.0% in the electrolyte. h) Raman spectra at the cathode‐electrolyte interface.

The unique surface properties of SPAN@QCS modify the electric double layer (EDL) and electrolyte solvation structure. With the positive charge for the QCS layer, its selective adsorption to PF_6_^−^ anions in the electrolyte was investigated. Molecular dynamics (MD) simulations reveal a significantly higher adsorption density of PF_6_^−^ on the QCS surface compared to the bare SPAN (**Figure** **2**a–c). The RDF profiles (Figure S4a, Supporting Information) show that the QCS surface exhibits a stronger primary coordination peak and slower decay, indicating enhanced PF_6_^–^ adsorption and a denser interfacial layer. The residence‐time distribution (Figure S4b, Supporting Information) shows longer PF_6_^–^ retention near QCS, suggesting a more stable interfacial structure. The adsorption energy for PF_6_^−^ on QCS (−560.8 kJ mol^−1^) further confirms its strong preferential adsorption (Figure 2d,e). Implicit solvent calculations (COSMO, ε = 35) reduce the adsorption energy but preserve the stronger QCS–PF_6_^–^ interaction. The adsorption energies for PF_6_^−^ on QCS and SPAN are −352.3 and −32.1 kJ mol^−1^, respectively. This strong QCS–PF_6_^−^ interaction was experimentally verified by ^19^F nuclear magnetic resonance (NMR) spectroscopy. A downfield shift of PF_6_^−^ peaks (Figure 2f) indicates reduced PF_6_^−^–solvent affinity and strong binding to QCS.^[^ ^46^, ^47^ ^]^ Zeta potential measurements (Figure 2g) show a higher positive value for SPAN@QCS‐1.0% (+2.06 mV) than SPAN (+0.71 mV), implying preferential accumulation of PF_6_^−^ anions within the inner Helmholtz plane (IHP) of SPAN@QCS‐1.0%. Raman spectra (Figure 2h) of soaked electrolytes exhibit peaks for free EC (ring bending, 720 cm^−1^) and free PF_6_^−^ (P–F symmetrical vibration, 741 cm^−1^).^[^ ^48^ ^]^ The intensity of free EC and PF_6_^−^ peaks are denoted as I_1_ and I_2_, respectively. Remarkably, the I_2_/I_1_ ratio of 0.52 for SPAN@QCS‐1.0% is significantly lower than that of 0.62 for blank electrolyte and 0.61 for SPAN, suggesting that partial PF_6_^−^ anions are adsorbed and bound by QCS. This further proves that QCS layer has stronger adsorption to PF_6_^−^ anions in the electrolyte. The anion‐dominated Helmholtz layer is demonstrated to promote the formation of inorganic‐rich interphase on SPAN surface and thus alters the CEI formation mechanisms, as discussed in the following section.

**Figure 3:**
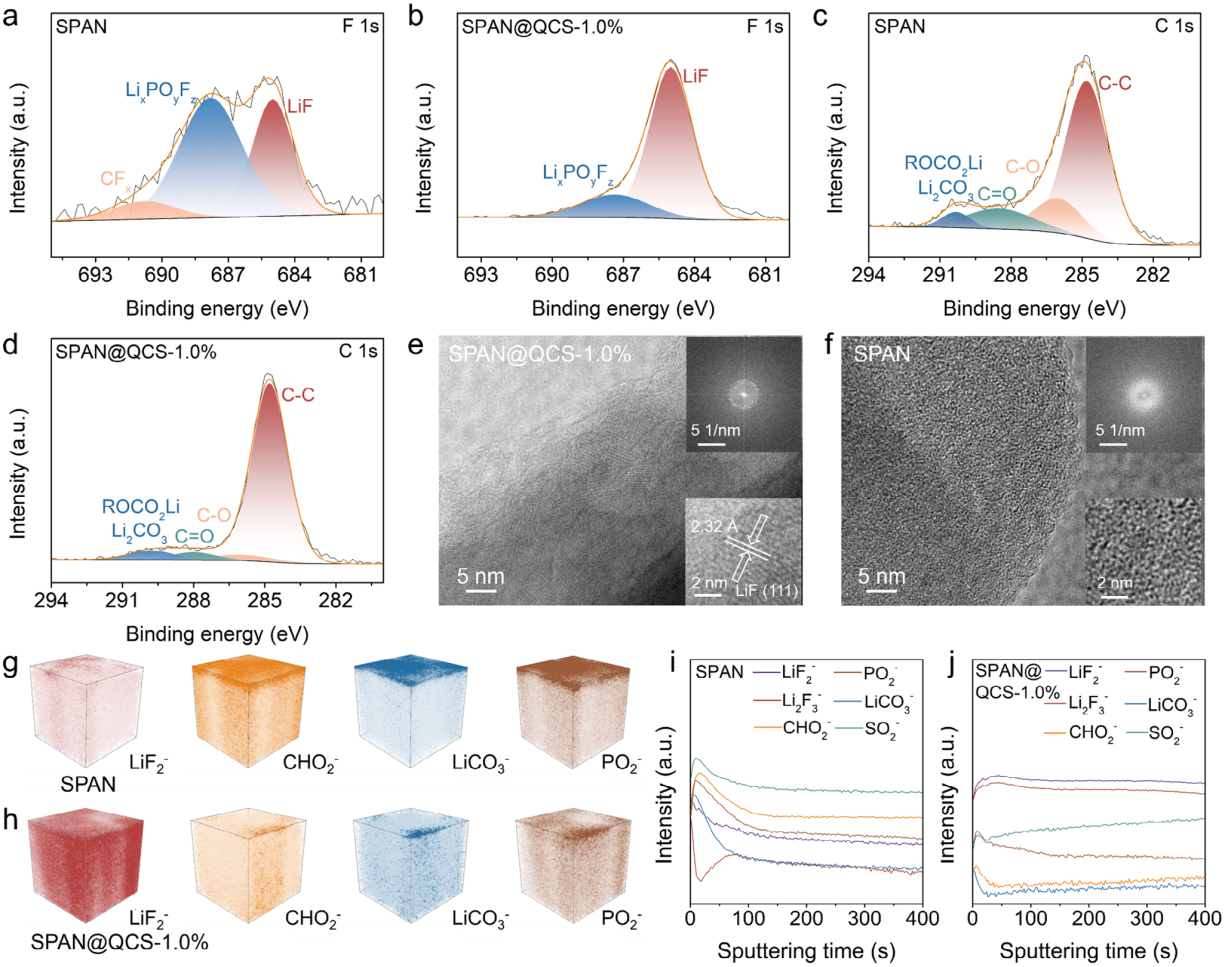
CEI layers characterization. a,b) F 1s XPS spectra for a) SPAN and b) SPAN@QCS‐1.0%. c,d) C 1s XPS spectra for c) SPAN and d) SPAN@QCS‐1.0%. e,f) HR‐TEM images of e) SPAN@QCS‐1.0% and f) SPAN. Insets of e,f) are the FFT patterns and lattice spacing of SPAN@QCS‐1.0% and SPAN cathodes, respectively. g,h) TOF‐SIMS spatial ion distribution maps for g) SPAN and h) SPAN@QCS‐1.0% cathodes. i,j) Depth profiles of i) SPAN and j) SPAN@QCS‐1.0% cathodes.

The chemical composition and distribution of the CEI layer on the cycled cathodes (0.2 C, 5 cycles) were then analyzed by XPS, HR‐TEM, and time‐of‐flight secondary ion mass spectrometry (TOF‐SIMs). F 1s XPS spectra of the SPAN cathode (**Figure** **3**a) reveal three deconvoluted peaks at 685.0, 687.8, and 690.9 eV, assigned to LiF, Li*_x_* PO*_y_* F*_z_*, and CF*_x_*, respectively.^[^ ^49^ ^]^ These species mostly originate from the extensive PF_6_^−^ and FEC decomposition. In contrast, the SPAN@QCS‐1.0% cathode (Figure 3b) exhibits only two F 1s peaks, corresponding to LiF and Li*_x_* PO*_y_* F*_z_*, primarily derived from PF_6_^−^ decomposition within the IHP of the EDL.^[^ ^50^ ^]^ Moreover, the LiF ratio reaches 80.47% (Figure S5, Supporting Information), indicating its predominance in the SPAN@QCS‐1.0% CEI. C 1s spectra of SPAN (Figure 3c) deconvolve into four peaks at 284.8, 286.0, 288.5, and 290.2 eV, assigned to C─C, C─O, C═O, and ROCO_2_Li/Li_2_CO_3_, respectively.^[^ ^51^, ^52^ ^]^ ROCO_2_Li/Li_2_CO_3_ arises from carbonate solvent decomposition and constitutes the main CEI component. Therefore, the peak area at 290.2 eV correlates with the extent of electrolyte decomposition. Notably, SPAN@QCS‐1.0% shows a significantly attenuated C 1s peak at 290.2 eV (Figure 3d), indicating superior suppression of carbonate solvent decomposition.

To confirm the effect of QCS on the CEI layer, control experiments were performed using an electrolyte without FEC (i.e., 1.0 m LiPF_6_ in EC/DEC). The XPS analysis of the cycled cathodes from these cells revealed that even in the absence of FEC, a substantial amount of LiF was detected in the F 1s spectrum of SPAN@QCS‐1.0% cathode. (Figure S6a,b, Supporting Information) Specifically, the LiF ratio of SPAN and SPAN@QCS‐1.0% cathode is 30.87% and 74.68%, respectively. This provides direct evidence that the decomposition of PF_6_^−^ anions, facilitated by their preferential adsorption/desolvation on our modified interface, is indeed a primary source of LiF. Similarly, the decomposition of carbonate solvents is effectively suppressed on the surface of the SPAN@QCS‐1.0% cathode (Figure S6c,d, Supporting Information).

HR‐TEM analysis is then performed to probe the CEI morphology. The cycled SPAN@QCS‐1.0% cathode (Figure 3e) displays distinct lattice fringes (≈2.32 Å spacing), indexed to the (111) planes of LiF. The corresponding FFT pattern further confirms LiF enrichment (Figure 3e inset). Conversely, the cycled SPAN cathode exhibits a disordered, amorphous CEI (Figure 3f), suggesting higher organic content. These results indicate that QCS coating enables the SPAN cathode to generate an inorganic CEI layer featuring extensive formation of LiF. TOF‐SIMS analysis further elucidated the CEI chemistry. Two‐dimensional (2D) planar distribution maps (Figure S7, Supporting Information) reveal congruent distribution profiles for LiF_2_^−^, CHO_2_^−^, PO_2_^−^, and LiCO_3_^−^ across the plane, indicating that the CEI composition is a composite of organic and inorganic phases. Compared with SPAN (Figure 3g and Figure S7a, Supporting Information), the cycled SPAN@QCS‐1.0% (Figure 3h and Figure S7b, Supporting Information) exhibits significantly stronger LiF_2_^−^ signals and weaker C_2_HO^−^/PO_2_^−^/LiCO_3_^−^ signals in both 2D and 3D visualizations. This demonstrates that the CEI components originate primarily from lithium salt decomposition, while carbonate solvent decomposition is effectively suppressed by the modified IHP structure. Notably, LiF is highly desirable in electrode‐electrolyte interphases owing to its high modulus, low Li^+^ diffusion barrier, and intrinsic electron‐insulating properties.^[^ ^53^ ^]^ Furthermore, LiF exhibits high ionic conductivity at grain boundaries when interfaced with other components (e.g., Li_2_O, Li_2_CO_3_, LiOH).^[^ ^54^ ^]^ Consequently, rapid Li^+^ transport can be achieved by the massively increased ionic carrier concentration at the electrode/electrolyte interface.

**Figure 4:**
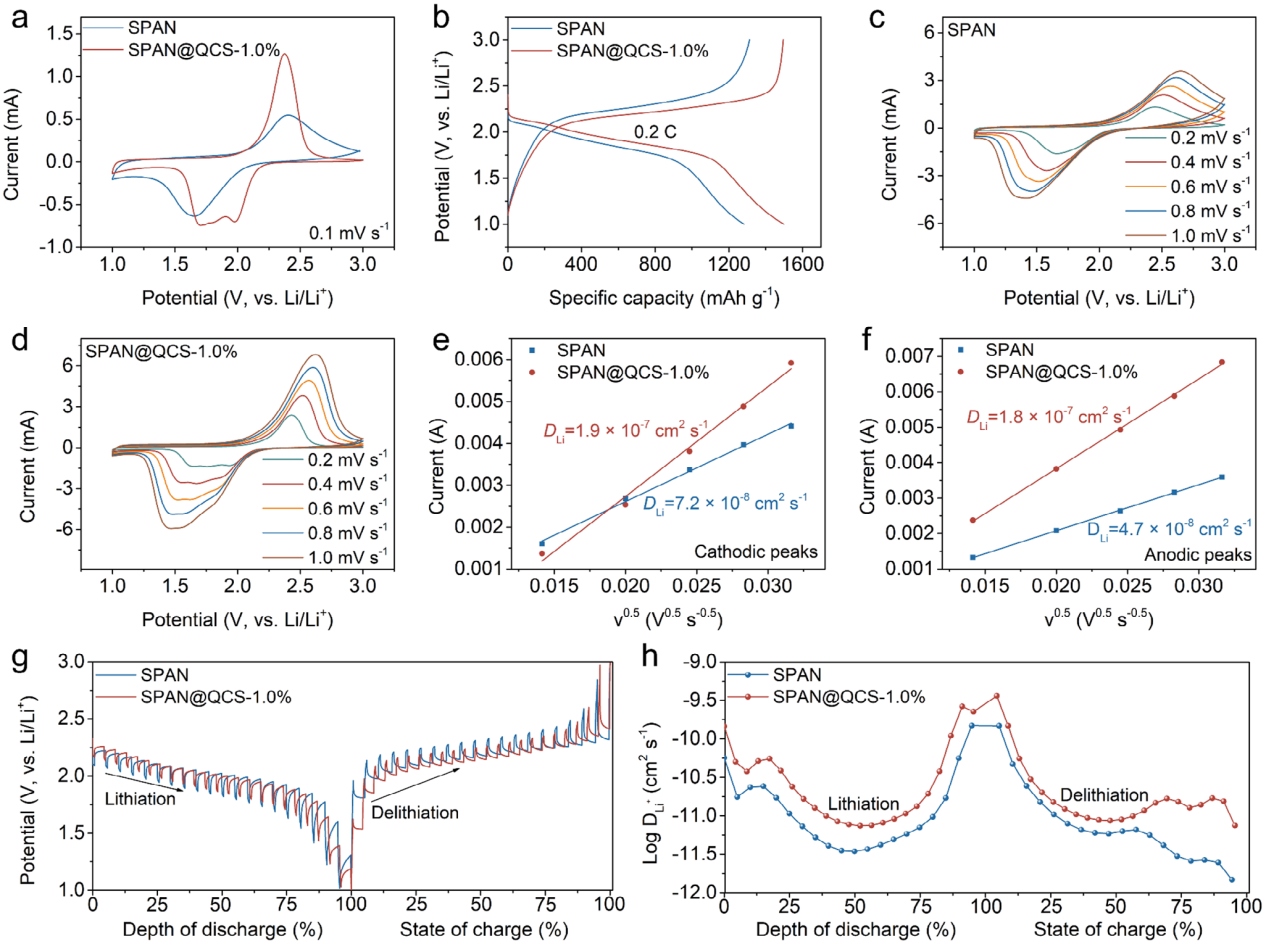
Characterization of electrochemical reaction kinetics. a) CV curves and b) charge–discharge curves of SPAN and SPAN@QCS‐1.0%. c,d) CV curves of c) SPAN and d) SPAN@QCS‐1.0% at different scan rates from 0.2‐1.0 mV s^−1^. e,f) Randles–Sevcik plot of peak current versus the square root of the scan rate‐derived *D*_Li_ values of SPAN and SPAN@QCS‐1.0% for e) discharge and f) charge processes. g) Voltage curves and h) Li^+^ diffusion coefficient of SPAN and SPAN@QCS‐1.0% obtained via the GITT technique.

The difference in electrochemical kinetics of SPAN and SPAN@QCS was investigated through cyclic voltammetry (CV) measurements at 0.1 mV s^−1^. As shown in **Figure** **4**a, the SPAN@QCS‐1.0% cathode exhibits a broad bimodal reduction signal at 1.98 and 1.70 V, indicating stepwise sulfur reduction to Li_2_S_2_ and Li_2_S, respectively.^[^ ^55^, ^56^ ^]^ In contrast, the SPAN cathode shows a single broad reduction peak at a lower potential, reflecting sluggish kinetics. During oxidation, SPAN@QCS‐1.0% displays significantly higher peak intensity than SPAN, confirming the enhanced electrochemical activity. Galvanostatic discharge–charge profiles at 0.2 C also demonstrate the advantage of QCS coating. SPAN@QCS‐1.0% delivers a high discharge capacity of 1499 mAh g^−1^ and an average discharge plateau of 1.9 V at 50% depth of discharge, exceeding SPAN in both metrics (Figure 4b). To further probe the electrochemical kinetics of SPAN@QCS‐1.0% cathode, CV measurements were performed with different scan rates from 0.2 to 1.0 mV s^−1^ (Figure 4c,d). The redox peak currents scale linearly with the square root of the scan rate (Figure 4e,f), indicating that the rate‐determining step is dominated by Li^+^ diffusion. Therefore, the Li^+^ diffusion coefficient can be calculated using the Randles–Ševčík equation:^[^ ^57^, ^58^ ^]^

(1) $$I_{p}=2.69\times 10^{5}\times n^{1.5}\times S\times D_{Li^{+}}^{0.5}\times v^{0.5}\times C_{Li^{+}}$$

where *I*_p_ represent the peak current (A), *n* represents the charge transfer number (*n* = 2 for Li–S batteries), *S* is the electrode area (cm^−2^), $D_{Li^{+}}$ is the Li^+^ diffusion coefficient, *v* is the scan rate (V s^−1^), and $C_{Li^{+}}$ refers to the concentration of Li^+^ (mol cm^−3^). Since *n*, *S*, $C_{Li^{+}}$ can be treated as constants, the slop between *I*_p_ and *v* ^0.5^ (the square roots of scan rates) reflect the $D_{Li^{+}}$. The $D_{Li^{+}}$ values for the reduction and oxidation peaks of the SPAN@QCS‐1.0% cathode are 1.9×10^−7^ and 1.8×10^−7^ cm^2^ s^−1^, respectively, both of which are higher than those of the pristine SPAN cathode. The electrodes with other QCS ratios (0.5 wt.% and 1.5 wt.%) also showed enhanced Li^+^ diffusion rates, but these improvements were slightly lower than that of SPAN@QCS‐1.0% due to the insufficient surface modification or increased impedance (Figure S8, Supporting Information). Moreover, galvanostatic intermittent titration technique (GITT) measurements showed that the SPAN@QCS‐1.0% cathode exhibits faster Li^+^ diffusion (Figure 4g,h). The fast Li^+^ diffusion is important for improving the rate capability of SPAN cells, as discussed in the electrochemical performance section.

**Figure 5:**
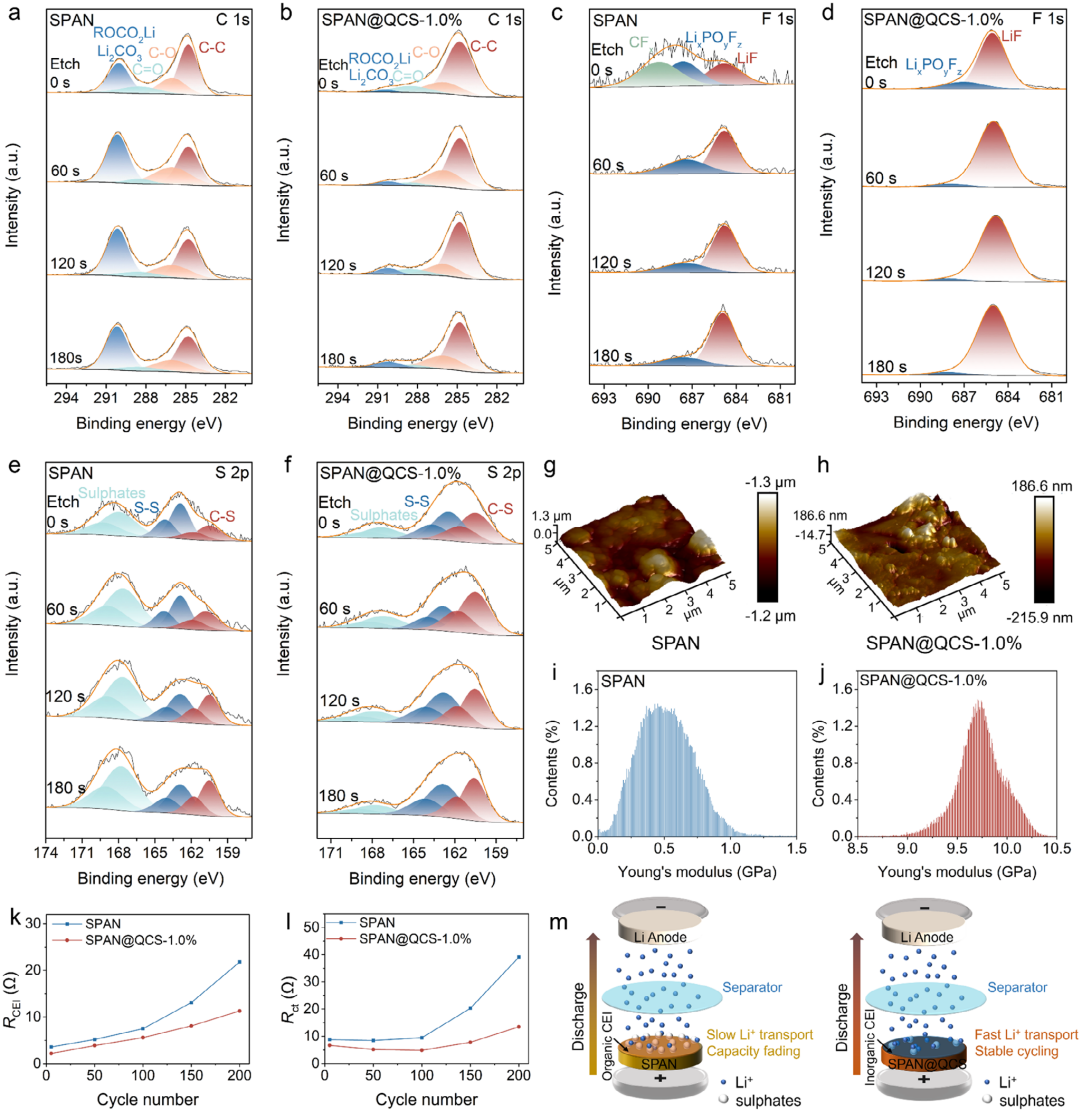
Interfacial stability characterization. a–f) In‐depth XPS spectra of SPAN and SPAN@QCS‐1.0% after 200 cycles. a,b) C1s spectra; c,d) F1s spectra; e,f) S 2p spectra. g,h) the AFM 3D images and i,j) Young's modulus distribution of SPAN and SPAN@QCS‐1.0% cathodes after 200 cycles. k) *R*_CEI_ and l) *R*_ct_ of SPAN and SPAN@QCS‐1.0%. m) Schematic illustration of the enhancement effect of LiF‐rich CEI on SPAN@QCS‐1.0% cathodes.

To investigate cathode interface stability, we performed XPS depth profiling on cycled cathodes (200 cycles). The C 1s spectra for SPAN (**Figure** **5**a) exhibit intense ROCO_2_Li/Li_2_CO_3_ signals at 290.2 eV, indicating significant carbonate solvent decomposition during cycling. In contrast, the SPAN@QCS‐1.0% cathode shows attenuated C 1s peak intensity at 290.2 eV across sputtering durations (Figure 5b), suggesting superior interfacial stability in suppressing carbonate solvent decomposition compared to baseline electrodes. Both SPAN and SPAN@QCS‐1.0% display LiF and Li*_x_* PO*_y_* F*_z_* peaks in their F 1s spectra. Additionally, the SPAN cathode exhibits intense CF*_x_* signals at 690.2 eV. We attribute the high Li*_x_* PO*_y_* F*_z_* and CF*_x_* content on SPAN to continuous electrolyte solvent reduction, resulting from a fragile and unstable CEI. As sputtering time increases from 0 to 60 s (Figure 5c), the CF_x_ signals diminish while the LiF ratio increases significantly, indicating an organic‐rich outer layer and inorganic‐rich inner layer characteristic of traditional electrode/electrolyte interphases.^[^ ^59^, ^60^ ^]^ For SPAN@QCS‐1.0%, the weaker Li*_x_* PO*_y_* F*_z_* and stronger LiF signals (Figure 5d) suggest the CEI primarily originates from PF_6_^−^ degradation within the IHP of the EDL. Increasing sputtering time (0 to 180 s) nearly eliminates Li*_x_* PO*_y_* F*_z_* signals, indicating a higher LiF proportion in the SPAN@QCS‐1.0% CEI than in SPAN. This LiF‐rich CEI effectively suppresses parasitic reactions between sulfur species and carbonate‐based electrolytes, enhancing interfacial electrochemical stability. S 2p XPS spectra (Figure 5e,f) reveal three characteristic doublets for both cathodes: C–S covalent bonds (161.7/162.9 eV), S–S bonds (163.5/164.7 eV), and sulfate species (168.3/169.5 eV).^[^ ^61^ ^]^ Crucially, SPAN@QCS‐1.0% exhibits significantly reduced sulfate signal intensity compared to SPAN, confirming that the LiF‐enriched inorganic CEI substantially mitigates interfacial side reactions between active materials and electrolytes.

The stability of QCS in the electrolyte during cycling was further evaluated through dissolution tests and post‐mortem EDX analysis. Dissolution tests confirmed that QCS is insoluble in carbonate electrolytes (Figure S9, Supporting Information). To further verify its electrochemical stability, cycled cells (after 200 cycles) were disassembled and examined by EDX on both the separator facing the cathode and the lithium metal anode. As shown in Figure S10a,b (Supporting Information), no nitrogen signal—a key elemental marker of QCS—was detected on either the separator or the lithium anode surface. These results strongly indicate that QCS does not undergo significant dissolution or migration into the electrolyte during cycling.

Atomic force microscopy (AFM) was employed to characterize the surface properties of SPAN and SPAN@QCS‐1.0% cathodes after 200 cycles (Figure 5g–j). As shown in Figure 5g, the bare SPAN cathode exhibits a thick surface layer, resulting from electrolyte solvent decomposition and parasitic reactions between active materials and the electrolyte. The low Young's modulus (≈0.5 GPa, Figure 5i) is consistent with a loosely arranged organic layer on the SPAN surface. In contrast, the SPAN@QCS‐1.0% cathode presents a smoother, more uniform surface with a Young's modulus of ≈10 GPa (Figure 5h,j), representing a twenty‐fold increase over bare SPAN. This enhancement is attributed to the selective adsorption of PF_6_^−^ anions by the QCS coating, which promotes the formation of a robust CEI. SEM was used to assess the structural integrity of cycled cathodes. The SPAN cathode developed extensive cracking after 200 cycles (Figure S11a, Supporting Information), indicative of significant structural degradation. The inherent CEI on SPAN fails to accommodate volume changes or suppress parasitic reactions, which will lead to severe capacity fade. Conversely, the SPAN@QCS‐1.0% cathode maintained structural integrity with minimal cracking (Figure S11b, Supporting Information), owing to the enhanced mechanical resilience of its LiF‐rich CEI. This stark contrast in electrode integrity confirms that the robust LiF‐enriched CEI effectively mitigates mechanical stress induced by volume expansion in SPAN@QCS‐1.0%.

The electrochemical stability of the electrode/electrolyte interface was further investigated by EIS measurements at different cycle intervals. The SPAN@QCS‐1.0% cathode exhibits lower impedance after 5 cycles (Figure S12a, Supporting Information), indicating the formation of a thin, conductive CEI layer. Nyquist plots for SPAN@QCS‐1.0% maintain consistent size and shape from 5 to 200 cycles, contrasting with significant changes observed for the SPAN cathode during cycling (Figure S12a–e, Supporting Information). All spectra display two semicircles and one low‐frequency slope. The high‐frequency semicircle corresponds to CEI film resistance (*R*_CEI_), the intermediate‐frequency semicircle to charge transfer resistance (*R*_ct_), and the low‐frequency slope to Warburg diffusion impedance.^[^ ^62^, ^63^ ^]^ Both *R*_CEI_ and *R*_ct_ values for SPAN@QCS‐1.0% show a smaller increase over 200 cycles (Figure 5k,l), demonstrating the advantage of the LiF‐enriched CEI in stabilizing the interface. Notably, *R*_ct_ decreases initially for both cathodes due to electrode activation, consistent with prior reports on SPAN‐based electrodes.^[^ ^64^, ^65^ ^]^ Figure 5m illustrates the enhancement mechanism of the SPAN@QCS‐1.0% cathode. The LiF‐rich inorganic CEI layer exhibits high mechanical strength and ionic conductivity, suppressing electrolyte‐active substance side reactions, which contributes to high‐rate capability and enhanced cycling stability.

**Figure 6:**
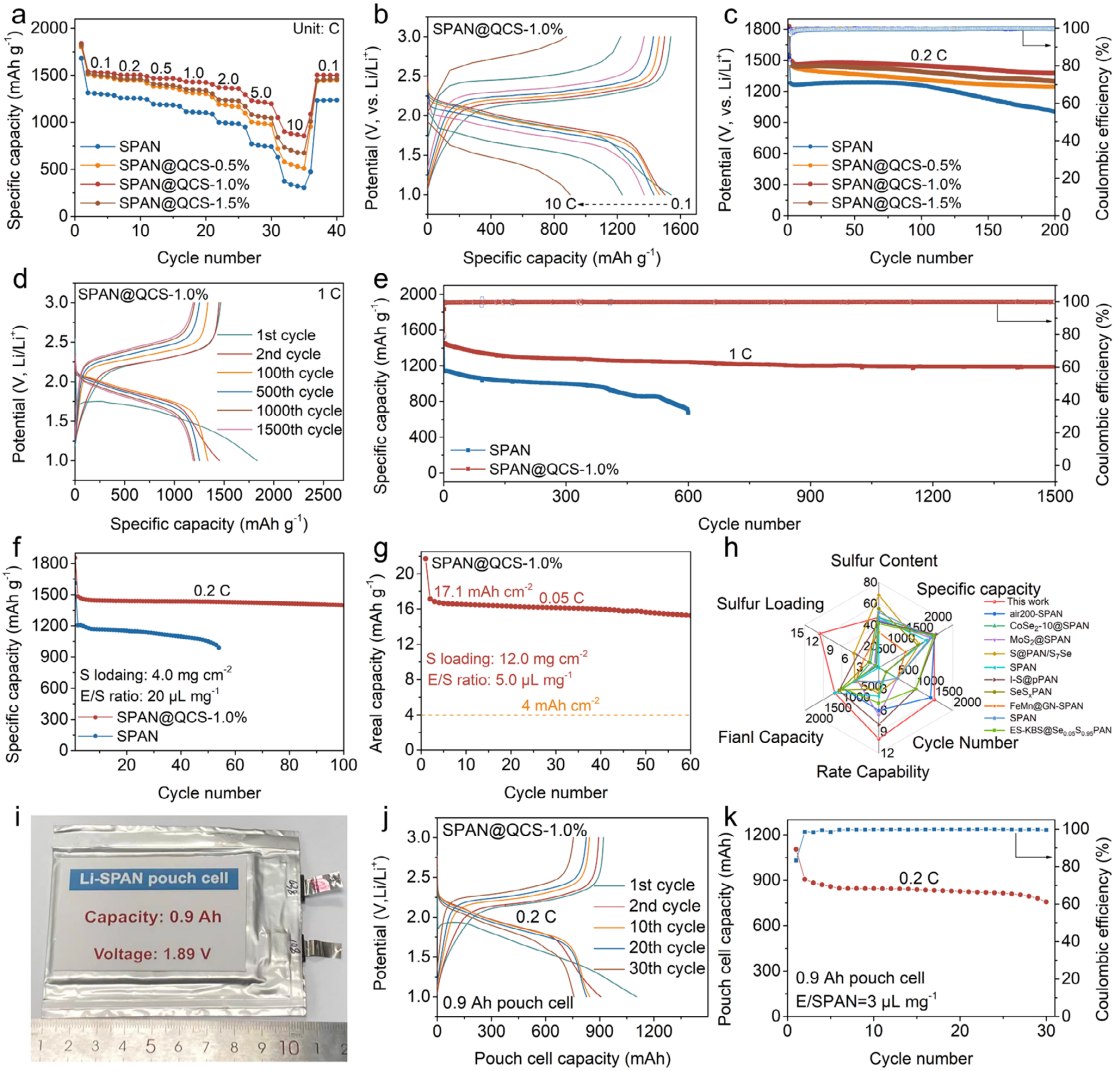
Electrochemical performance. a) Rate performance of SPAN and SPAN@QCS‐x%. b) Charge–discharge profiles of SPAN@QCS‐1.0% at different current rates. c) Cycling stability of SPAN and SPAN@QCS‐x% at 0.2 C. d) Charge–discharge profiles of SPAN@QCS‐1.0% at selected cycles. e) Cycling stability of SPAN and SPAN@QCS‐1.0% at 1 C. f) Cycling stability of SPAN and SPAN@QCS‐1.0% cathodes under high mass loading (4.0 mg cm^−2^). g) Cycling stability and areal capacity of SPAN@QCS‐1.0% cathode under practical conditions: high mass loading (12.0 mg cm^−2^) and low E/S ratio (5.0 µL mg^−1^). h) Performance comparison between SPAN@QCS‐1.0% cathode and others advanced SPAN electrode systems in Table S2, Supporting Information). i) Optical photograph, j) charge–discharge profiles, and k) cycling stability of SPAN@QCS‐1.0% pouch cell. The specific capacity and current rate are calculated based on the weight of sulfur in the cathode materials.

The electrochemical performance of SPAN and SPAN@QCS‐x% was evaluated in CR2025 coin cells using galvanostatic measurements. As shown in **Figure** **6**a,b, the SPAN@QCS‐1.0% cathode exhibits the highest reversible capacities of 1542, 1503, 1468, 1433, 1371, and 1232 mAh g^−1^ at 0.1, 0.2, 0.5, 1.0, 2.0, and 5.0 C, respectively. Even at an ultra‐high rate of 10 C, it maintains a high discharge capacity of 902 mAh g^−1^, demonstrating excellent rate capability. In contrast, the SPAN cathode delivers only 374 mAh g^−1^ at 10 C, corresponding to a low capacity utilization of 22.3%. Cycling performance at 0.2 C (Figure 6c) shows the SPAN@QCS‐x% cathode outperforming SPAN in both stability and specific capacity. SPAN@QCS‐1.0% and SPAN deliver initial reversible capacities (second cycle) of 1499 and 1282 mAh g^−1^, respectively, retaining 92.0% and 78.5% of capacity after 200 cycles. The SPAN@QCS‐0.5% and SPAN@QCS‐1.5% cathodes also show good cycle performance, with capacity retention rates of 85.2% and 87.7%, respectively. This performance difference is attributed to QCS modifying the structure of the EDL and CEI on the SPAN surface. The SPAN@QCS‐1.0% cathode also demonstrates stable cycling at higher rates (Figure 6d,e). Under extended cycling at 1 C, it delivers discharge capacities of 1454 and 1189 mAh g^−1^ after 2 and 1500 cycles, respectively, corresponding to an extremely low fading rate of 0.012% per cycle. In contrast, the pristine SPAN electrode suffered accelerated degradation, retaining only 59.4% of its initial capacity (1135 to 675 mAh g^−1^) after 600 cycles.

Furthermore, SPAN@QCS‐1.0% cathodes paired with ether electrolytes also demonstrated excellent electrochemical performance, including high reversible capacity (1491 mAh g^−1^ at 0.2 C; 1431 mAh g^−1^ at 1 C) and good cycling stability (87.0% retention over 200 cycles at 0.2 C; 82.0% retention over 300 cycles at 1 C) (Figure S13, Supporting Information). This suggests that the CEI engineering induced by QCS provides universality across different electrolyte systems. Post‐mortem XPS analysis confirmed this hypothesis (Figure S14, Supporting Information). The SPAN@QCS‐1.0% cathode cycled in the ether‐based electrolyte reveals a CEI layer rich in LiF species derived from TFSI^−^ decomposition, along with a hybrid organic‐inorganic interface similar to that observed in carbonate electrolytes. This consistent CEI composition confirms that the QCS strategy effectively constructs stable interfaces regardless of the electrolyte solvent environment. More importantly, the LiF‐rich CEI layer suppresses polysulfide dissolution and interfacial side reactions, resulting in a significant reduction of sulfate species on the cathode surface. This mechanism fundamentally explains the stable cycling performance of SPAN@QCS‐1.0% cathodes in ether‐based electrolytes.

To evaluate SPAN@QCS‐1.0% for practical applications, we fabricated thick cathodes with varying sulfur loadings and performed galvanostatic charge–discharge testing. As shown in Figure 6f, the SPAN@QCS‐1.0% cathode with a sulfur loading of 4.0 mg cm^−2^ delivered a high discharge capacity of 1484 mAh g^−1^ at 0.2 C, comparable to thin electrode performance. After 100 cycles, a high reversible capacity of 1398 mAh g^−1^ is retained, corresponding to a high capacity retention of 94.2%. Enhanced electrochemical kinetics also conferred good rate performance (Figure S15a,b, Supporting Information): capacities of 1521, 1472, 1405, 1346, 1272, and 1118 mAh g^−1^ were achieved at 0.1 to 1.0 C, respectively. Upon returning to 0.1 C, the capacity recovered to 1479 mAh g^−1^, demonstrating excellent stability. With increasing sulfur loading, SPAN@QCS‐1.0% maintained robust electrochemical performance (Figure S16, Supporting Information). It delivered excellent reversible capacities of 1483 (4.2 mg cm^−2^), 1406 (6.0 mg cm^−2^), and 1338 (7.8 mg cm^−2^) mAh g^−1^ at 0.2 C, corresponding to high areal capacities of 6.3, 8.4, and 10.4 mAh cm^−2^, respectively. After cycling for 50 cycles, reversible capacities of 1424, 1349, and 1218 mAh g^−1^ with high retentions of 96.0%, 96.0%, and 91.0% can still be maintained. Even under harsher conditions (12.0 mg cm^−2^ loading, electrolyte/sulfur ratio = 5 µL mg^−1^), the cathode delivered a high initial areal capacity of 21.7 mAh cm^−2^ and an outstanding reversible capacity of 17.1 mAh cm^−2^—exceeding typical commercial Li‐ion battery areal capacities (≈4 mAh cm^−2^) by more than fourfold (Figure 6g). To our knowledge, the comprehensive electrochemical performance of SPAN@QCS‐1.0% exceeds representative literature values (Figure 6h and Table S2, Supporting Information).

Moreover, we assembled an Ah‐level pouch cell using SPAN@QCS‐1.0% as active cathode materials to evaluate the viability of surface QCS coating in practical conditions (Figure 6i–k). The detailed parameters (e.g., negative/positive ratio, number of stacked layers, cell capacity, average discharge voltage) are listed in Table S3 (Supporting Information). The pouch cell shows typical voltage profiles of SPAN and stable cycling over 30 cycles, suggesting the beneficial effects of QCS coating on stabilizing SPAN cells. Although its energy density (175 Wh kg^−1^, including package) remains suboptimal, continued optimization of the pouch cell design is expected to lead to a further increase in energy density. In addition, the cycle life of pouch cells is primarily limited by the unstable lithium anode. Protecting the lithium anode is expected to result in a longer cycle life in the future. Collectively, these results demonstrate SPAN@QCS‐1.0%'s significant potential for practical Li–S batteries.

## 3 Conclusion

In summary, QCS was employed as an additive to stabilize the CEI layer on SPAN cathodes. The abundant quaternary ammonium groups in QCS modify the SPAN surface properties via selectively adsorbing PF_6_^–^ anions from the electrolyte. This structure promotes the formation of an anion‐derived CEI enriched with LiF species. The robust LiF‐rich CEI facilitates rapid Li^+^ transport and significantly suppresses detrimental side reactions between the cathode and electrolyte. Consequently, the SPAN@QCS‐1.0% cathode delivers a reversible specific capacity of 1499 mAh g^−1^ at 0.2 C and exhibits excellent rate capability (902 mAh g^−1^ at 10 C). It also demonstrates outstanding long‐term cycling stability, retaining 81.8% of its capacity after 1500 cycles at 1 C. Notably, under high sulfur loading and lean electrolyte conditions, the cathode achieves an exceptional areal capacity of 17.1 mAh cm^−2^ with stable cycling over 60 cycles. The Ah‐level pouch‐cell further demonstrates the viability of the QCS coating strategy in practical cells. In the future, the exploration of advanced SPAN cathodes with higher sulfur content, guided by stable and conductive CEI design, will further enhance the energy density and application prospects of Li–S batteries.

## 4 Experimental Section

### Synthesis of SPAN

Sublimed sulfur (> 99.99%, Aladdin) and PAN (M_W_ = 150000, Aladdin) were used as received. QCS (degree of substitution = 98%, Shanghai Macklin Biochemical Co., Ltd.) and PAA (Guangdong Canrd New Energy Technology Co., Ltd.) were purchased commercially. SPAN composites were synthesized by heating a mixture of sulfur and PAN (mass ratio 6:1) at 330 °C for 6 h under an argon atmosphere. This sulfur excess ensured saturated sulfur incorporation. Subsequently, excess surface sulfur was removed by heating the product at 300 °C for 2 h under flowing argon. After cooling to room temperature, the resulting powder was ground to yield SPAN.

### Materials Characterization

The morphology and structure of materials were characterized using Field‐emission SEM (Gemini SEM500) and HR‐TEM (JEM‐F200). Elemental distribution was analyzed by energy‐dispersive X‐ray spectroscopy (EDS, UltimMax 100) coupled to the HR‐TEM. XPS was performed using a Gammadata‐Scienta SES 2002 analyzer with a monochromic Al Kα source (1486.6 eV). XRD patterns were acquired on a D8 DISCOVER diffractometer (Bruker) using Cu Kα radiation (λ = 1.5418 Å). Elemental (C, H, N, S) content was determined using an elemental analyzer (vario EL cube). Raman spectroscopy was performed on a Renishaw in Via microscope with a 0532 nm excitation laser. FTIR spectra were recorded on a Nicolet iS50 spectrometer (Thermo Fisher Scientific). Electrolyte solvation structure was investigated by solution‐state NMR spectroscopy (Bruker AVANCE III HD 500 MHz) using dimethyl sulfoxide‐d_6_ (DMSO‐d_6_) as the solvent. Zeta potential measurements were conducted using a Zeta sizer Nano ZSP (Malvern Instruments, Worcestershire, UK). Electrode morphology and Young's modulus were characterized by AFM (Bruker Dimension ICON).

### Electrode Preparation and Electrochemical Tests

SPAN@QCS‐x% cathodes were fabricated by homogenizing SPAN, PAA binder, CNTs, and QCS additive (80‐x:10:10:x wt. ratio) in water. The resulting slurry was stirred for 6 h, doctor‐blade coated onto Al foil, and vacuum‐dried at 60 °C for 10 h. Cathode discs (10 mm diameter) were punched from the dried electrode film. Control SPAN cathodes were prepared identically, omitting QCS. CR2025 coin cells were assembled in an argon‐filled glovebox. Lithium foil served as the anode, Celgard 2325 trilayer microporous membrane as the separator, and 1 m LiPF_6_ in ethylene carbonate/diethyl carbonate (EC/DEC, 1:1 v/v) with 10 wt.% fluoroethylene carbonates (FEC) as the electrolyte. The ether electrolyte consisted of 1 m LiTFSI and 1 wt% LiNO_3_ in 1,2‐dimethoxyethane (DME)/1,3‐dioxolane (DOL) (1:1 by volume). All electrochemical measurements were performed at 25 °C. Unless otherwise specified, the active material mass loading was 1.0–1.2 mg cm^−2^ and the electrolyte‐to‐sulfur ratio was ≈30 µL mg^−1^. CV tests were conducted using an electrochemical workstation (CHI760e, CH Instruments) in the voltage range of 1.0 to 3.0 V (vs Li/Li^+^). Galvanostatic cycling for stability and rate performance assessment was performed using a Neware battery test system (Shenzhen Neware, China) within the same voltage range (1.0–3.0 V). EIS measurements were acquired with a CHI660e electrochemical workstation (CH Instruments), applying a 5 mV AC amplitude across a frequency range of 100 kHz to 0.01 Hz. GITT measurements were carried out on the Neware system between 1.0 and 3.0 V. A constant current pulse of 0.1 C (1 C = 1675 mA g^−1^) was applied for 30 min to record the closed‐circuit voltage, followed by a 2 h relaxation period to attain the quasi‐open‐circuit voltage; this sequence was repeated continuously. For the Ah‐level pouch cell, the SPAN loading and mass fraction were 4.2 mg cm^−2^ and 85 wt%, respectively, and the thickness of the lithium anode was 90 µm. The cathode size was 5×7.5 cm, and the number of cathodes and anodes was 4 and 5 in each cell, respectively.

### Molecular Dynamics Simulations

All MD simulations were carried out using the COMPASS II force field in the Forcite module of Materials Studio. An amorphous cell with dimensions of 45 × 25 × 35 Å^3^ was constructed, containing 30 PF_6_^–^ ion pairs and a mixture of EC/DEC solvent molecules. The system was equilibrated under the NVT ensemble at 298 K with a 1 fs time step.

Trajectory data from multiple time windows were used to calculate averaged structural distributions and statistical deviations. RDFs and residence‐time distributions were analyzed to evaluate the adsorption and dynamic behavior of PF_6_^–^ near the SPAN and QCS surfaces. Implicit solvent calculations were further performed using the COSMO model (ε = 35, corresponding to EC/DEC) to account for dielectric screening effects. The adsorption energy (*E*_ads_) between the PF_6_^–^ ion and the surface was calculated using the following equation:

(2) $$E_{\mathrm{ads}}=E_{\mathrm{complex}}-\left(E_{\mathrm{surface}}+E_{\mathrm{ion}}\right)$$

where *E*_complex_ is the total potential energy of the combined system, and *E*_surface_ and *E*_ion_ represent the potential energies of the isolated surface and the single PF_6_^–^ ion, respectively. A more negative *E*_ads_ indicates stronger adsorption. Additionally, the density distribution of PF_6_^–^ along the Z‐direction was extracted from the equilibrated trajectories to analyze the depth and extent of ion adsorption on each surface.

### Statistical Analysis

No formal statistical testing was applied, as the consistent performance trends across replicates are the primary indicator of material performance. For electrochemical measurements, the galvanostatic charge–discharge tests were performed on at least three independently fabricated cells (n ≥ 3) for each type of electrode to ensure the reproducibility of the results. The data presented in the main figures are from a single representative measurement unless otherwise stated. The mean value and standard deviation of the specific capacity and retention are presented in Table S4–S8 (Supporting Information).

## Conflict of Interest

The authors declare no conflict of interest.

## Supporting information



Supporting Information

## Data Availability

The data that support the findings of this study are available from the corresponding author upon reasonable request.
