# Supplementary material for: Cathode Electrolyte Interphase Engineering by Quaternized Chitosan for Stabilized Li–SPAN Batteries
Source: Adv Sci (Weinh). 2025 Nov 21;13(8):e15551. doi: 10.1002/advs.202515551 (PMC12884775; doi:10.1002/advs.202515551)
Supplement: Supplementary file 1 — Supporting Information [file ADVS-13-e15551-s001.docx]

Supporting information

**Cathode Electrolyte Interphase** **Engineering by** **Quaternized Chitosan for Stabilized Li–SPAN Batteries**

Runhe He, Hao Liu, Qing Gao, Dong Cai, Kuikui Xiao, Yinhang Zhang, Xinmin Zhang, Xianyang Cheng, Yuhui Wang, Lixing Kang, Huagui Nie*, Yatao Liu*, Zhi Yang*

Dr. R. He, Q. Gao, Dr. D. Cai, Dr. K. Xiao, Dr. Y. Zhang, Prof. H. Nie, Prof. Z. Yang

Key Laboratory of Carbon Materials of Zhejiang Province, Wenzhou University, Wenzhou, 325035, China

E-mail: huaguinie@126.com; yang201079@126.com

Dr. H. Liu

College of Materials Science and Engineering, University of Jinan, Jinan, 250022, China

X. Zhang, X. Cheng, Y. Wang, Prof. Y. Liu

State Key Laboratory of Organic-Inorganic Composites, College of Chemical Engineering, Beijing University of Chemical Technology, Beijing 100029, China

E-mail: [liuyatao@buct.edu.cn](mailto:liuyatao@buct.edu.cn)

Dr. R. He, Prof. L. Kang

Key Laboratory of Multifunctional Nanomaterials and Smart Systems, Division of Advanced Materials, Suzhou Institute of Nano-Tech and Nano-Bionics, Chinese Academy of Sciences, Suzhou 215123, China

**Keywords:** cathode electrolyte interphase; interface engineering; lithium−sulfur battery; quaternized chitosan; sulfurized polyacrylonitrile


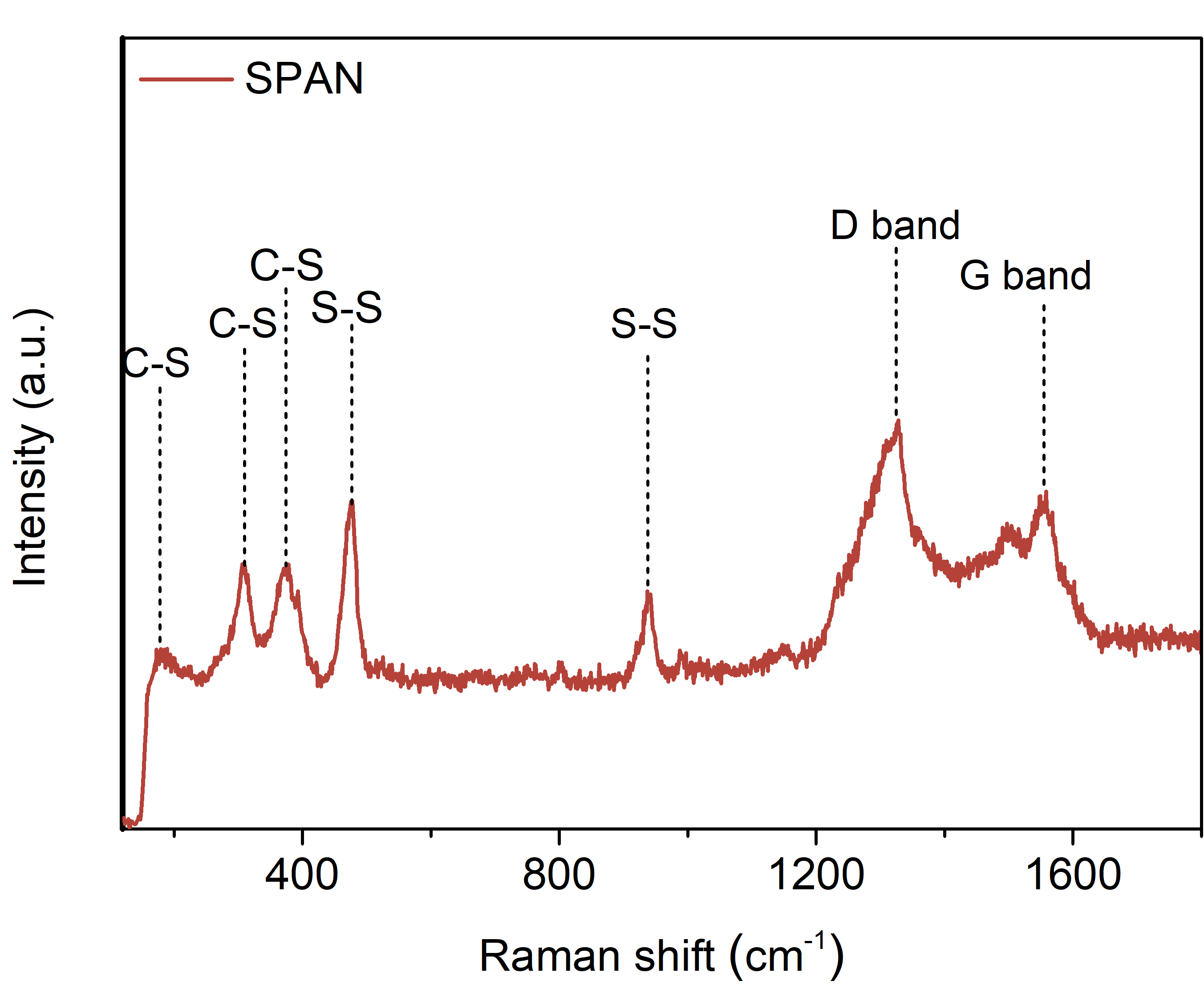


**Figure S1.** Raman spectra of SPAN.


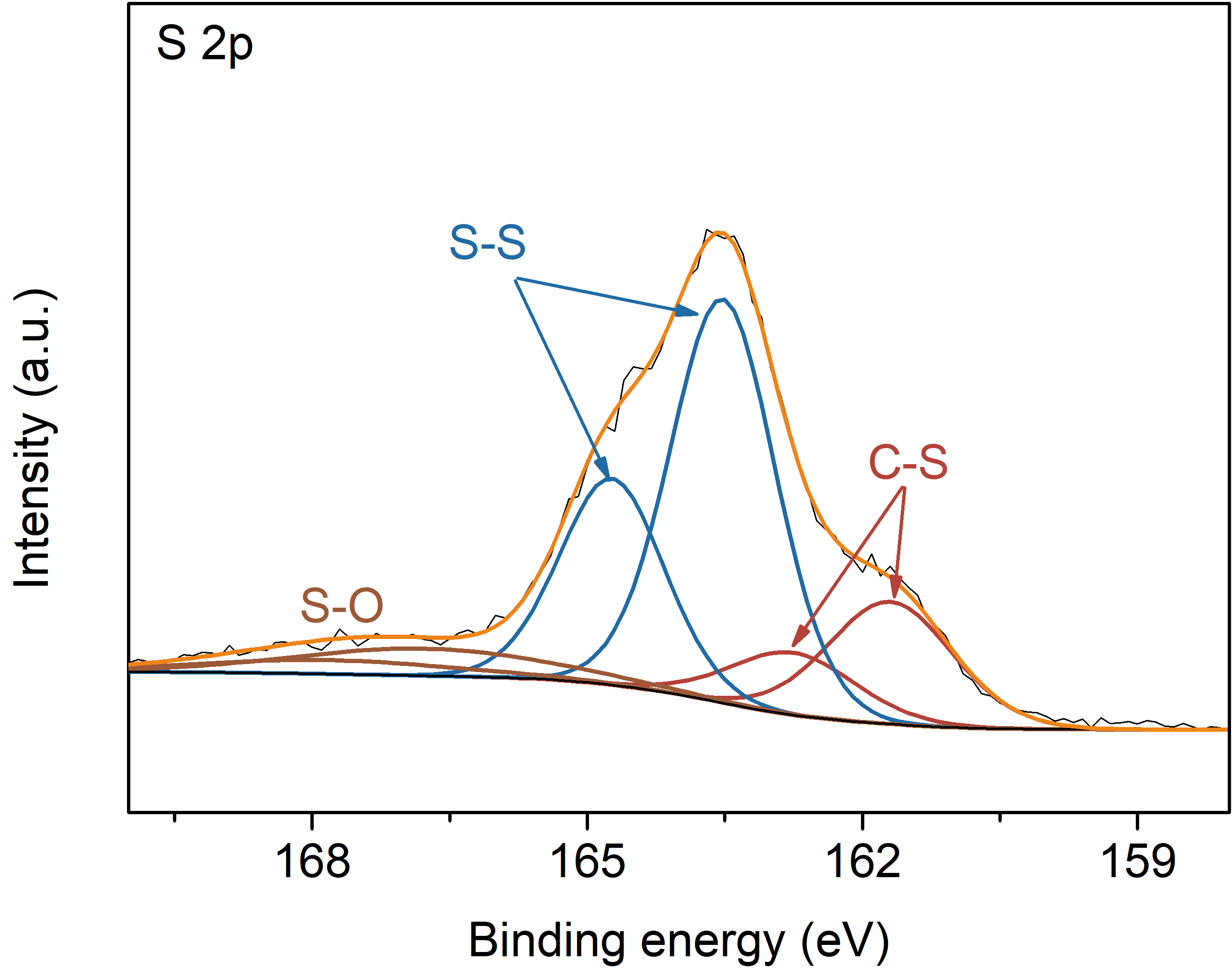


**Figure S2.** S 2p XPS spectrum of SPAN.

**Table S1.** The C, N, H, and S content for SPAN based on the elemental analysis.

| Material | C (%) | N (%) | H (%) | S (%) |
| --- | --- | --- | --- | --- |
| SPAN | 38.20 | 13.61 | 1.19 | 47.00 |


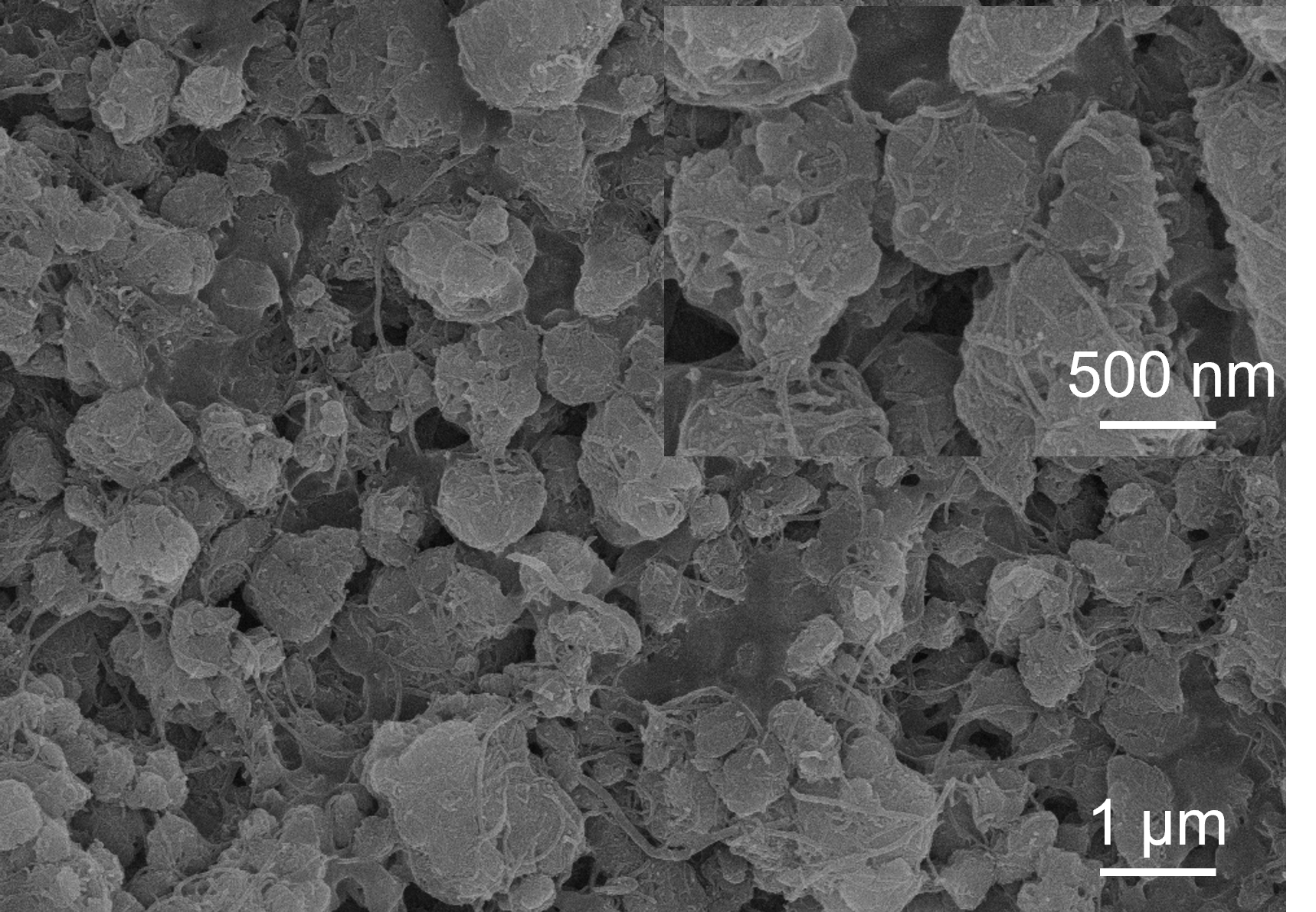


**Figure S3.** The SEM image of SPAN@QCS-1.0% cathode.


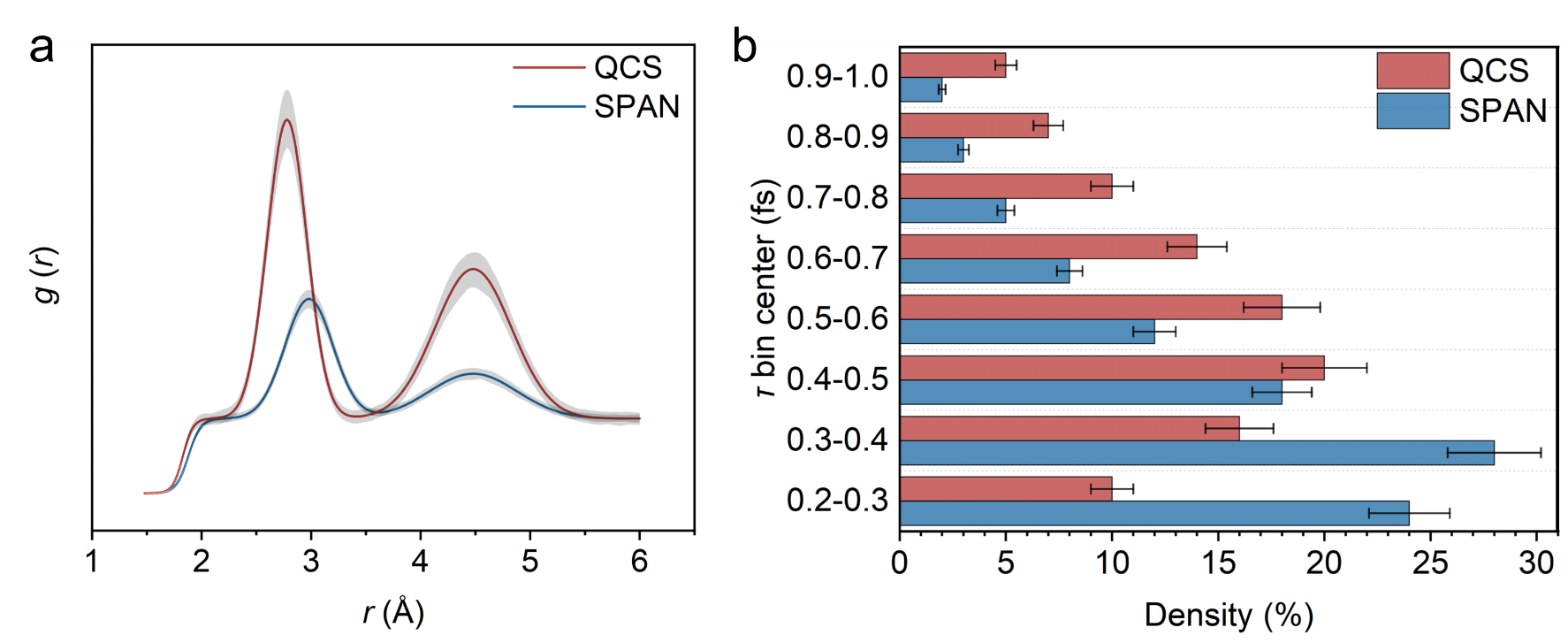


**Figure S4.** a) RDF profiles of PF_6_^−^ around surface C atoms on QCS and SPAN. b) Residence-time distributions of PF_6_^−^ near the surface.


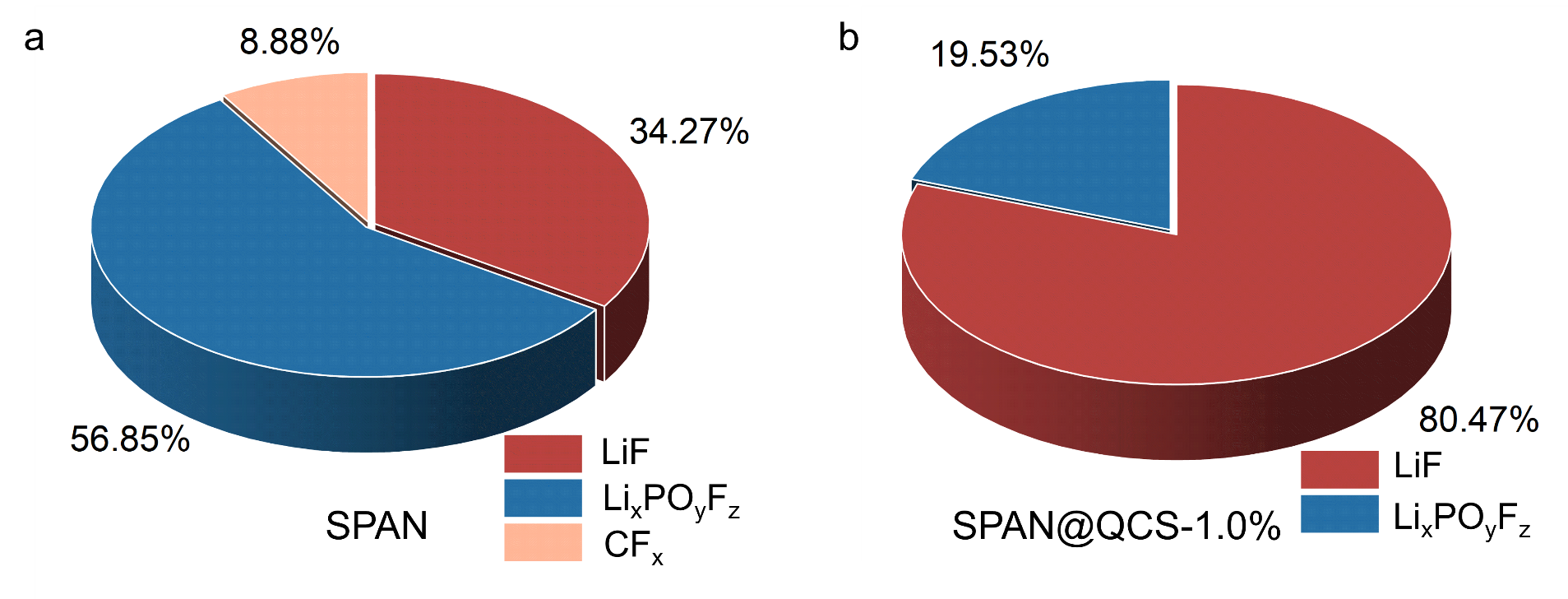


**Figure S5.** The LiF ratio of different cathodes obtained from XPS analysis results: a) SPAN and b) SPAN@QCS-1.0%.


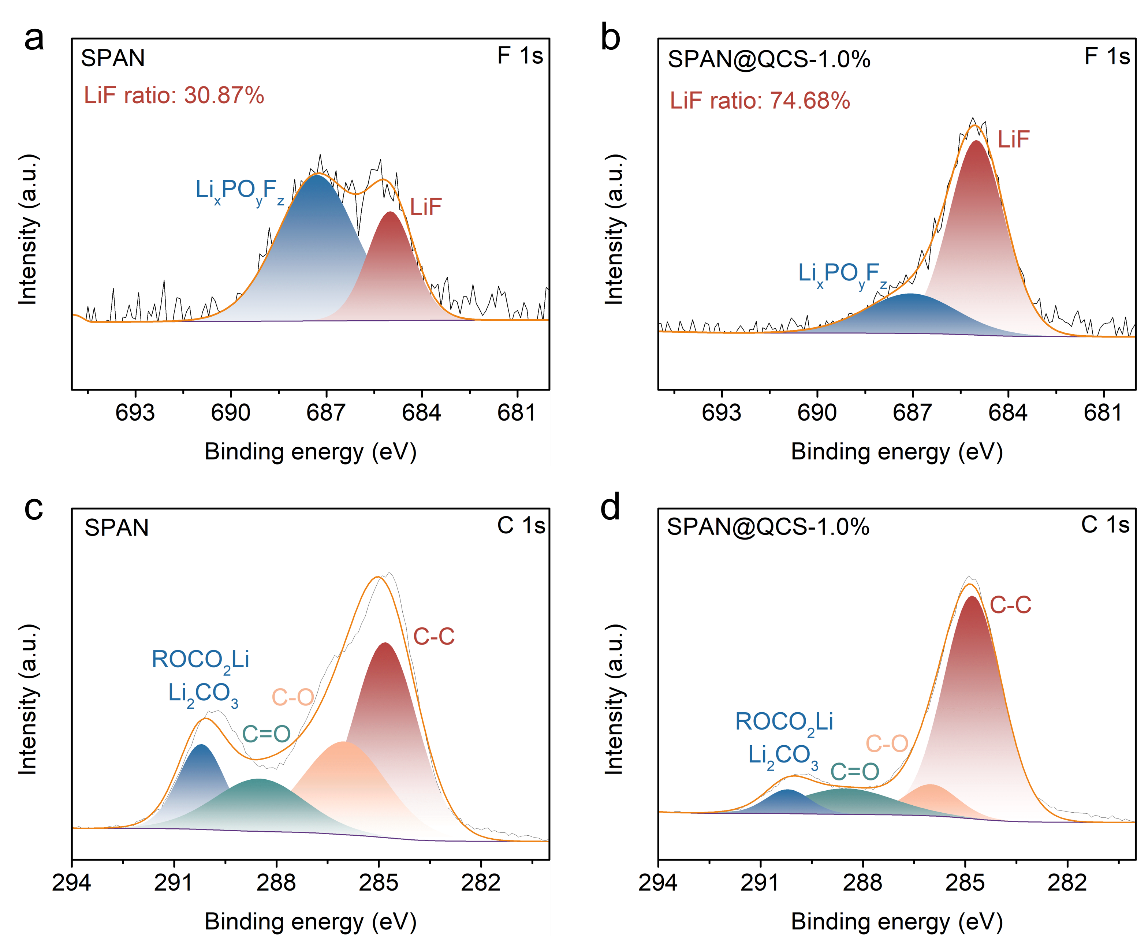


**Figure S6.** XPS analysis of SPAN-based cathodes in carbonate electrolyte without FEC. a, b) F 1s of a) SPAN and b) SPAN@QCS-1.0%. c, d) C 1s of c) SPAN and d) SPAN@QCS-1.0%.


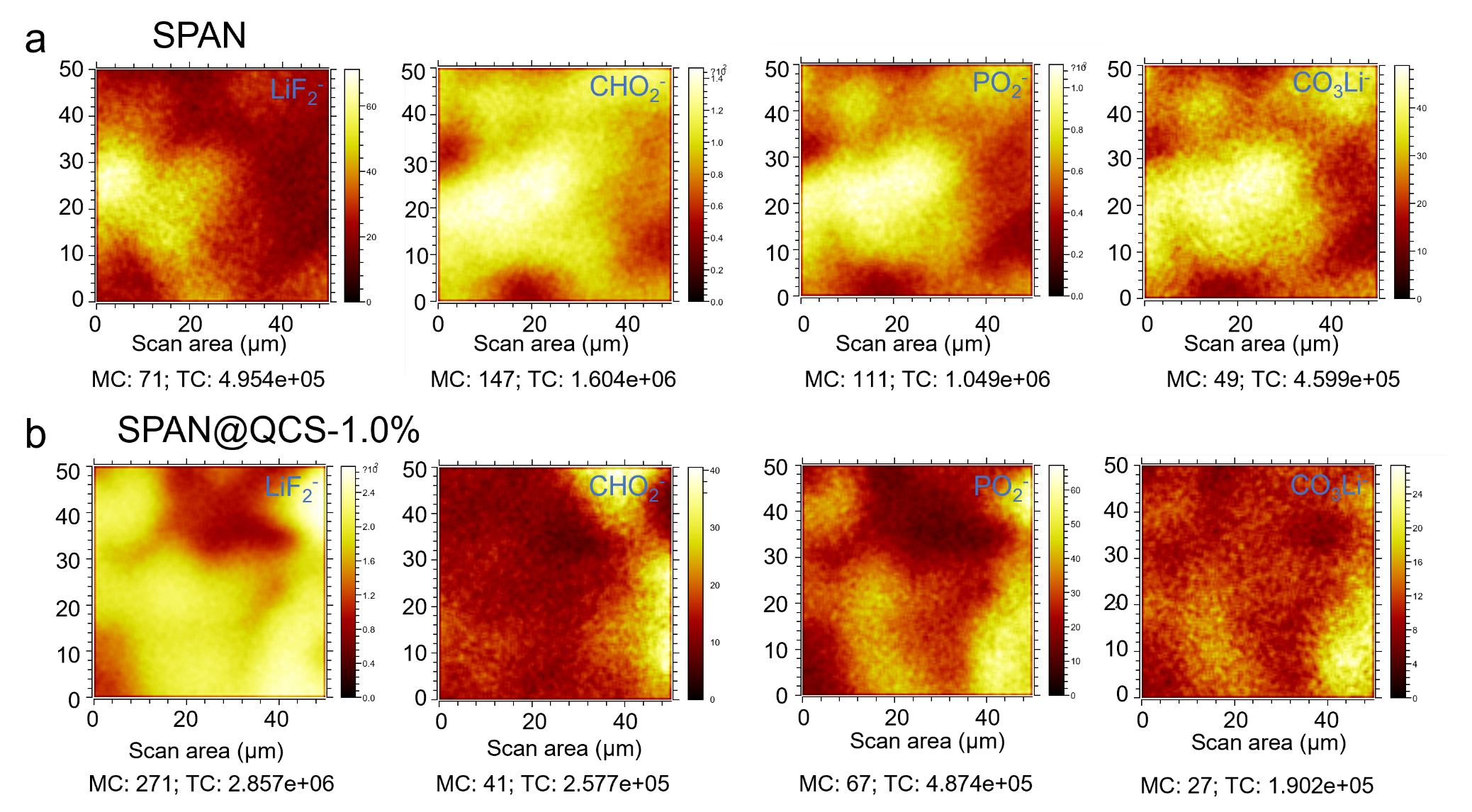


**Figure S7.** The 2D ions distribution map of a) SPAN and b) SPAN@QCS-1.0% cathodes detected by TOF-SIMS. Note that MC represents the maximum counts, and TC means the total intensity of secondary ions.


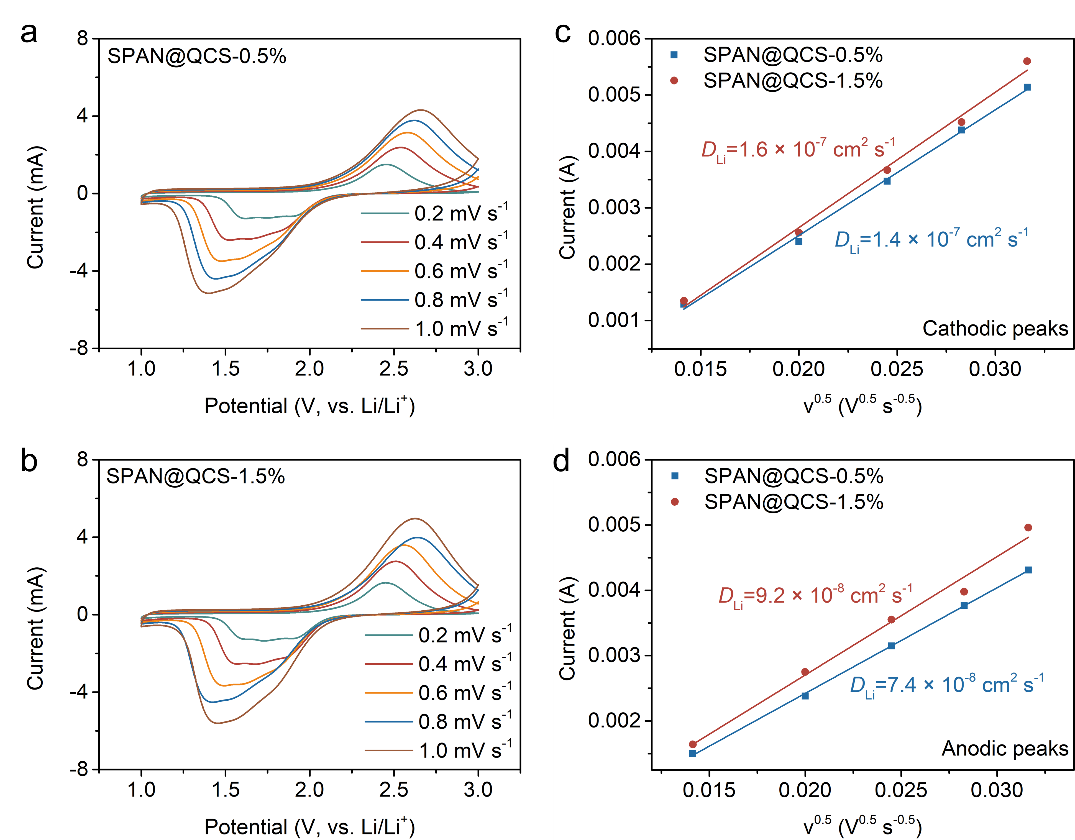


**Figure S8.** a) CV curves of SPAN@QCS-0.5% and SPAN@QCS-1.5% at different scan rates from 0.2-1.0 mV s^−1^. c, d) Randles–Sevcik plot of peak current versus square root of the scan rate-derived *D*_Li_ values of SPAN@QCS-0.5% and SPAN@QCS-1.5% for c) discharge and d) charge processes.


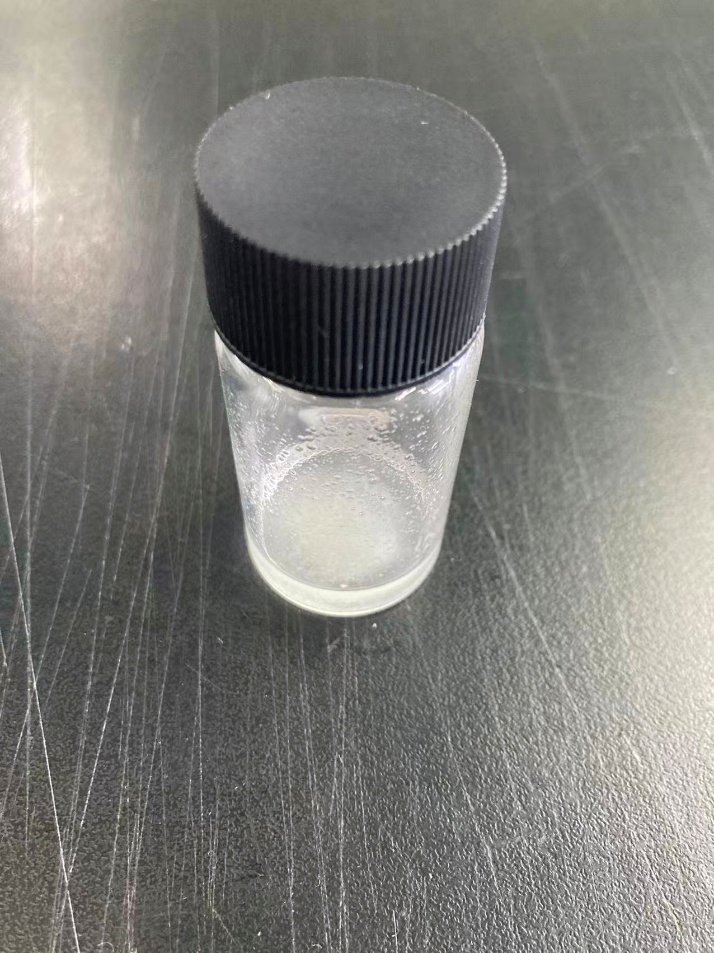


**Figure S9.** Digital photograph of the mixture containing quaternized chitosan (QCS, 30 mg) and electrolyte (3 mL) after 20 minutes of sonication.


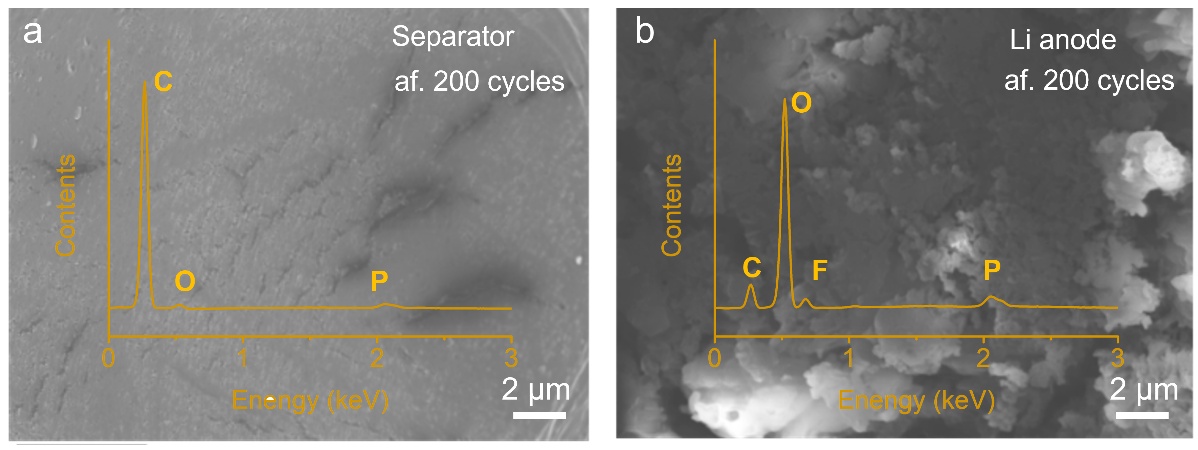


**Figure S10.** SEM images of a) separator and b) Li anode disassembled from the SPAN@QCS-1.0% cells after 200 cycles. Insets of a) and b) are EDX plots of separator and Li anode, respectively.


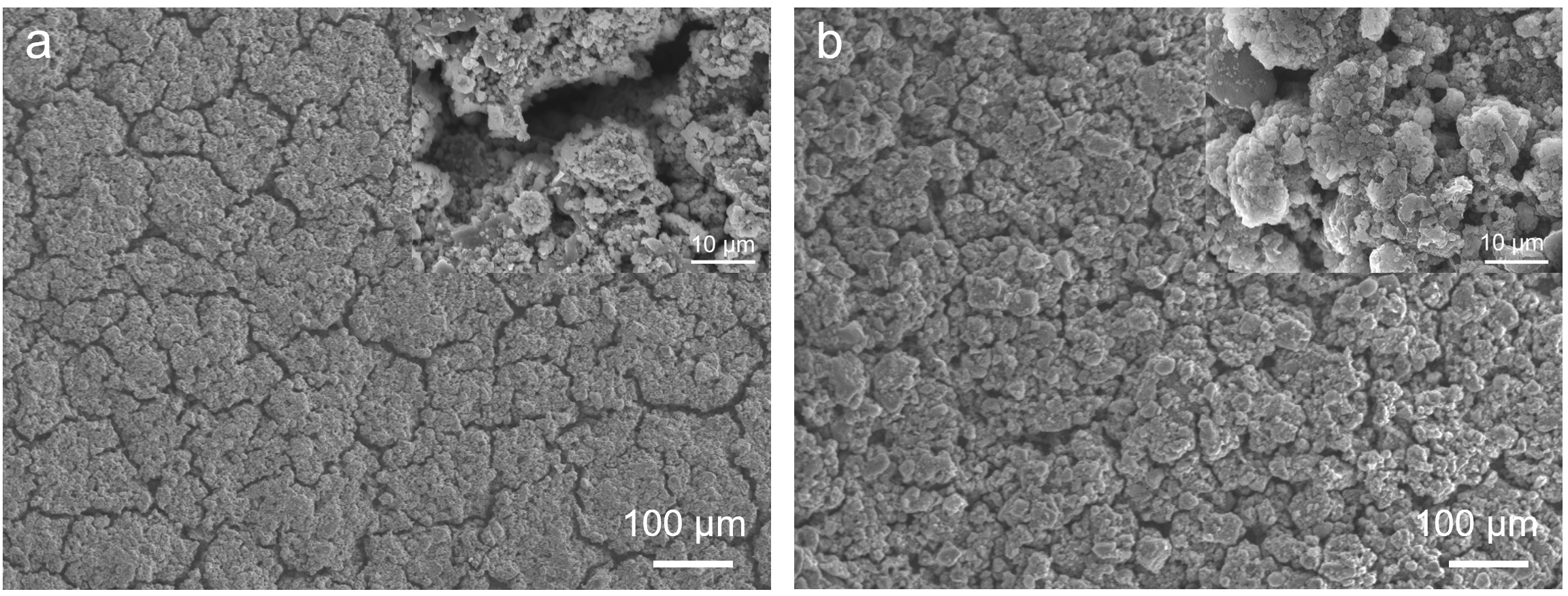


**Figure S11.** The SEM images of a) SPAN and b) SPAN@QCS-1.0% cathodes after cycling for 200 cycles.


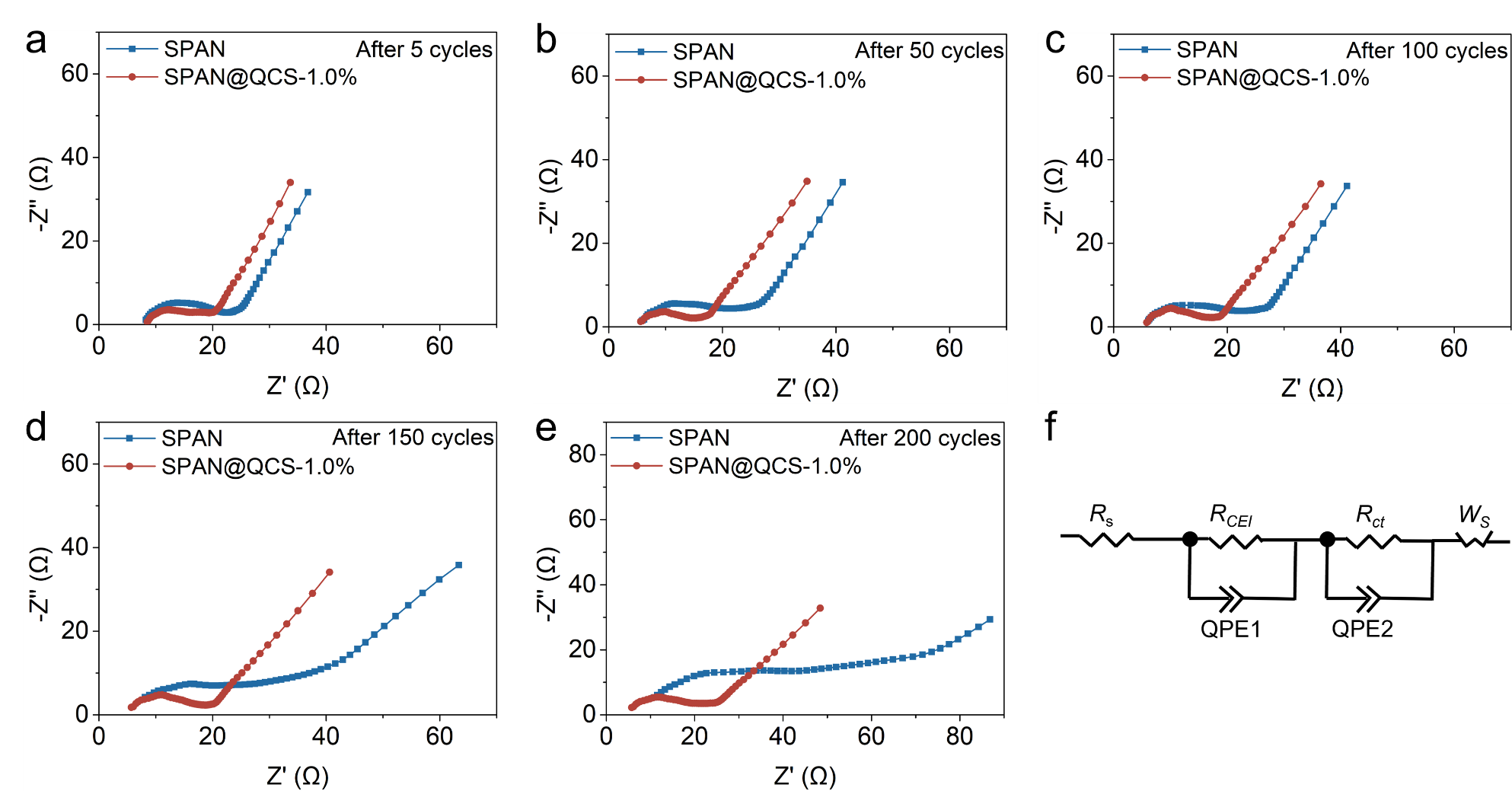


**Figure S12.** EIS plots of SPAN and SPAN@QCS-1.0% cathodes after a) 5, b) 50, c) 100, d) 150, and e) 200 cycles. f) equivalent circuit.


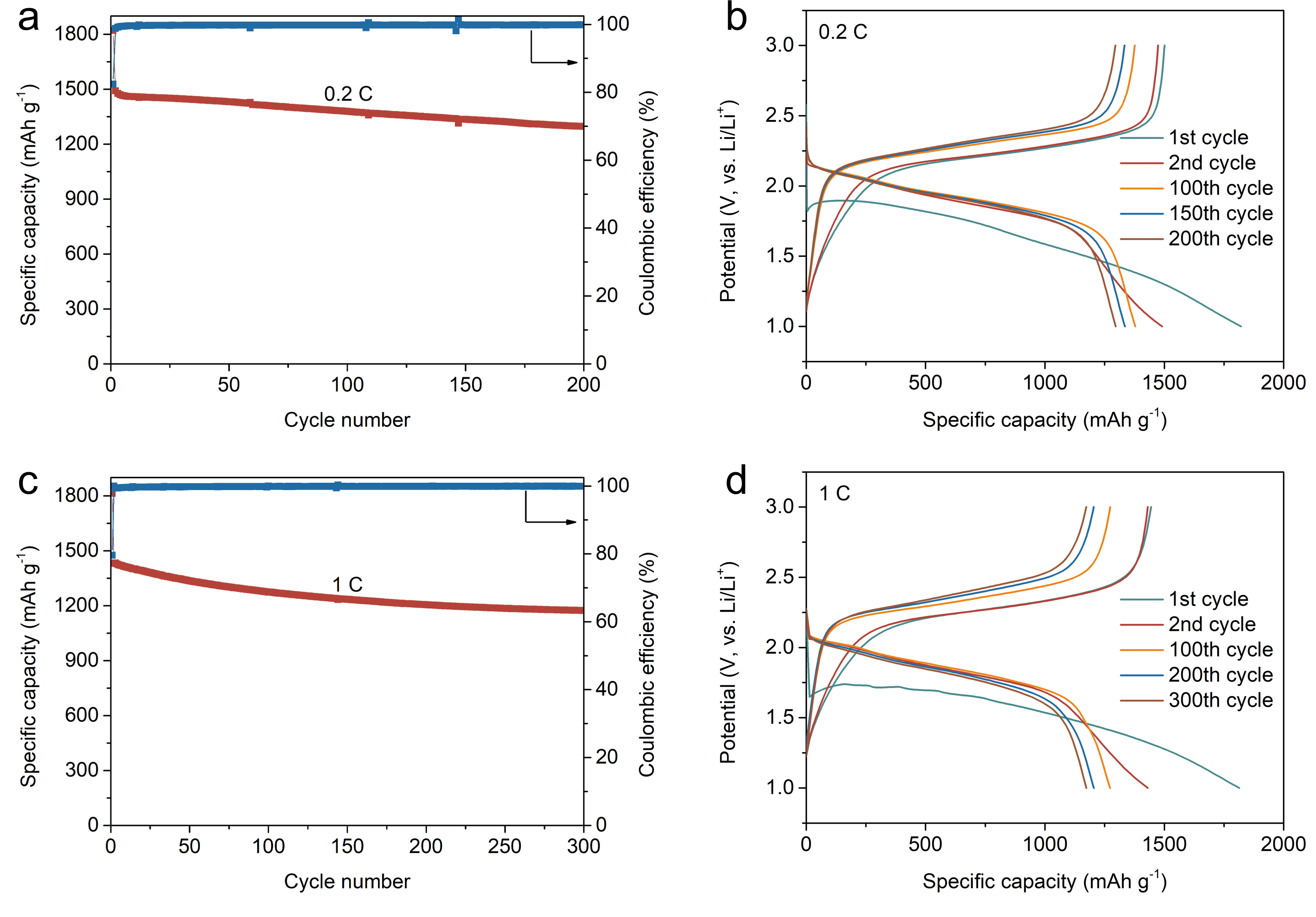


**Figure S13.** The electrochemical properties of SPAN@QCS-1.0% cathode in ether electrolyte. a) Cycling performance at 0.2 C and b) corresponding charge-discharge curves. c) Cycling performance at 1 C and d) corresponding charge-discharge curves.


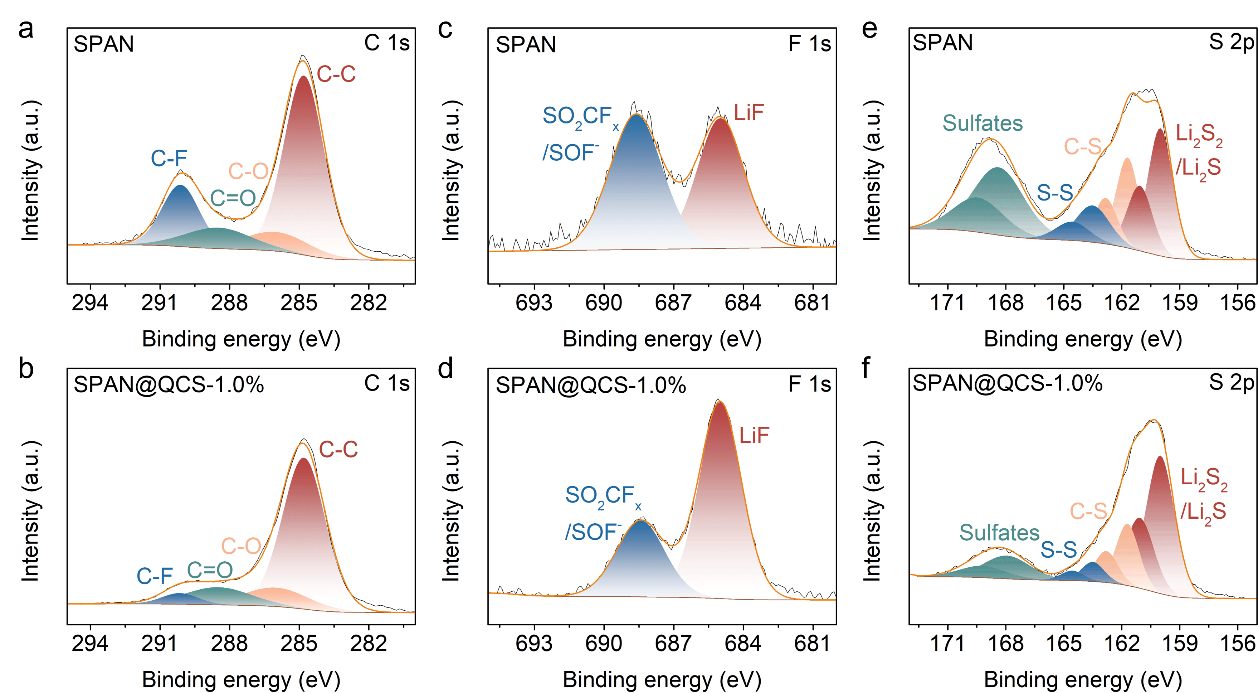


**Figure S14.** XPS analysis of CEI layer on SPAN and SPAN@QCS-1.0% cathodes in ether-based electrolyte. a, b) C 1s spectra of a) SPAN and b) SPAN@QCS-1.0 cathodes. c, d) F 1s spectra of c) SPAN and d) SPAN@QCS-1.0% cathodes. e, f) S 2p spectra of e) SPAN and f) SPAN@QCS-1.0% cathodes.


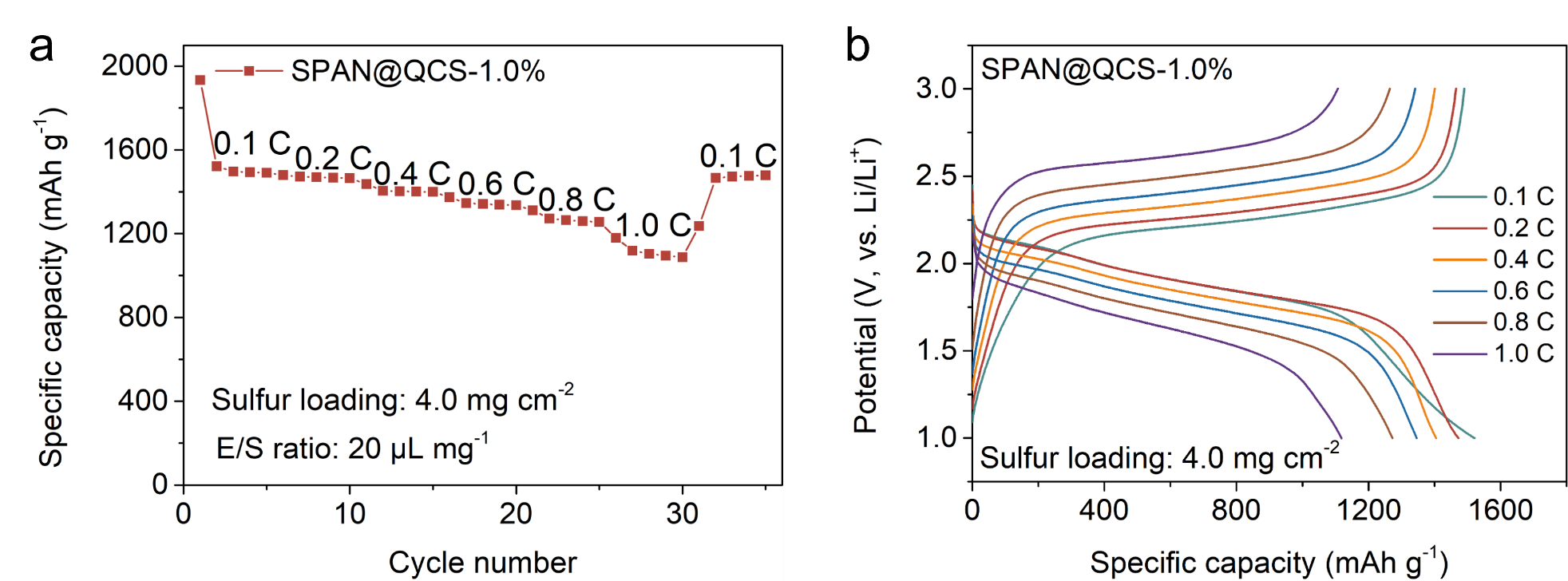


**Figure S15.** a) Rate performance of SPAN@QCS-1.0% with sulfur loading of 4.0 mg cm^-2^ and b) corresponding charge-discharge curves.


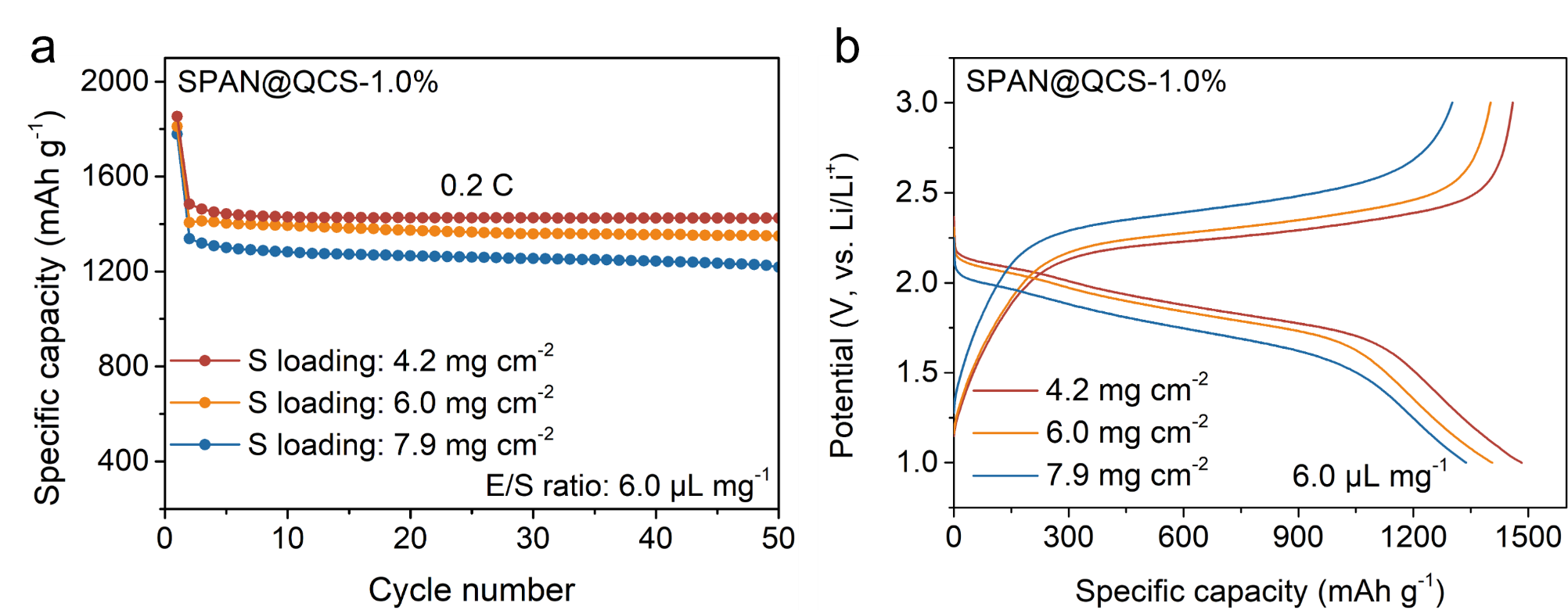


**Figure S16.** a) Cycle performance and b) Galvanostatic charge-discharge profiles of SPAN@QCS-1.0% cathode with sulfur loading of 4.2, 6.0, and 7.9 mg cm^−2^.

**Table S2.** The performance comparison of this work with some similar composite. Battery testing is conducted in a similar electrolyte system.

| Materials ^Reference^ | Ratio of active material (wt%) | Areal loading (mg cm^-2^) | Capacity retention (mAh g^-1^) | Rate capacity (mAh g^-1^) |
| --- | --- | --- | --- | --- |
| air200-SPAN ^[1]^ | 43.64 | 0.44 | 978 (3 C, 1400 cycles) | 1102 (6 C) |
| CoSe_2_-10@SPAN ^[2]^ | 47.1 | 1.67 | 675 (0.63 C, 500 cycles) | N/A |
| MoS_2_@SPAN ^[3]^ | 44.9 | 0.90 | 588 (2.7 C, 500 cycles) | 715 (6.7 C) |
| S@PAN/S_7_Se ^[4]^ | 68 | 5.0 | 648 (1.2 C, 500 cycles) | 667 (3.6 C) |
| SPAN ^[5]^ | 46.73 | N/A | 1181 (0.2 C, 200 cycles) | 170 (4 C) |
| I-S@pPAN ^[6]^ | 42.49 | 4.3 | 1077 (2 C, 1000 cycles) | 605 (8 C) |
| SeS*_x_*PAN ^[7]^ | 55 | 2.0 | 1055 (0.2 C, 200 cycles) | 545 (3 C) |
| FeMn@GN-SPAN ^[8]^ | 33.2 | 3.0 | 623 (0.3 C, 500 cycles) | 303 (2 C) |
| SPAN ^[9]^ | 51.7 | 0.72 | 638 (1 C, 500 cycles) | 1048 (2 C) |
| ES-KBS@Se_0.05_S_0.95_  PAN ^[10]^ | 41.2 | 0.6 | 966 (2 C, 1000 cycles) | 1130 (5 C) |
| SPAN@QCS-1.0% (This work) | 47.0 | 12.0 | 1189 (1 C, 1500 cycles) | 1232 (5 C)  902 (10 C) |

**Table S3.** Pouch cell parameters and energy density.

| Parameters | Value |
| --- | --- |
| Areal SPAN loading | 4.2 mg cm^-2^ |
| Thickness of Li foil | 90 µm |
| Electrode size | 5 × 7.5 cm |
| Negative/positive ratio | 5.1 |
| Number of cathode layer | 4 |
| Number of anode layer | 5 |
| Electrolyte/SPAN ratio | 3 µL mg^-1^ |
| Cell capacity | 907 mAh |
| Average discharge voltage | 1.89 V |
| Cell energy | 1.71 Wh |
| Cell weight | 9.8 g |
| Cell energy density (including package) | 175 Wh kg^-1^ |

**Table S4.** The rate performance of SPAN and SPAN@QCS-1.0% cathodes. All data were obtained from at least three independently repeated batteries (n ≥ 3).

| Rate | SPAN | SPAN@QCS-1.0% |
| --- | --- | --- |
| 0.1 C (mAh g^-1^) | 1315±10 | 1541±11 |
| 0.2 C (mAh g^-1^) | 1261±9 | 1501±10 |
| 0.5 C (mAh g^-1^) | 1193±9 | 1465±10 |
| 1.0 C (mAh g^-1^) | 1115±7 | 1436±9 |
| 2.0 C (mAh g^-1^) | 1102±11 | 1374±10 |
| 5.0 C (mAh g^-1^) | 771±12 | 1230±12 |
| 10 C (mAh g^-1^) | 376±14 | 906±18 |

**Table S5.** The capacity and capacity retention of SPAN@QCS-1.0% cathode. All data were obtained from at least three independently repeated batteries (n ≥ 3).

| SPAN@QCS-1.0% | Reversible capacity (mAh g^-1^) | Capacity retention (mAh g^-1^) |
| --- | --- | --- |
| 0.2 C | 1496±10 | 1378±12, 200 cycles |
| 1.0 C | 1456±9 | 1186±15, 1500 cycles |

**Table S6.** The capacity and capacity retention of SPAN cathode. All data were obtained from at least three independently repeated batteries (n ≥ 3).

| SPAN | Reversible capacity (mAh g^-1^) | Capacity retention (mAh g^-1^) |
| --- | --- | --- |
| 0.2 C | 1285±9 | 1003±11, 200 cycles |
| 1.0 C | 1138±8 | 671±14, 600 cycles |

**Table S7.** The capacity and capacity retention of SPAN and SPAN@QCS-1.0% cathodes at a high sulfur loading of 4.0 mg cm^−2^. All data were obtained from at least three independently repeated batteries (n ≥ 3).

| Specimen | Reversible capacity (mAh g^-1^) | Capacity retention (mAh g^-1^) |
| --- | --- | --- |
| SPAN | 1206±10 | 1050±12, 50 cycles |
| SPAN@QCS-1.0% | 1486±11 | 1394±13, 100 cycles |

**Table S8.** The areal capacity and capacity retention of SPAN@QCS-1.0% cathode at a high sulfur loading of 12.0 mg cm^–2^ and low E/S ratio of 5 µL mg^–1^. All data were obtained from at least three independently repeated batteries (n ≥ 3).

| Specimen | Areal capacity (mAh cm^-2^) | Capacity retention (mAh cm^-2^) |
| --- | --- | --- |
| SPAN@QCS-1.0% | 17.2±0.2 | 15.3±0.3, 60 cycles |

**References**

[1] J. Wang, Z. Du, G. Lv, X. Zhao, C. Li, X. Chen, Y. Huang, Enhancing the backbone regularity of sulfurized polyacrylonitrile for long-life Li-SPAN batteries, *J. Mater. Chem. A* **2025,** 13, 21545.

[2] Z.-Q. Xu, R. Zou, W.-W. Liu, G.-L. Liu, Y.-S. Cui, Y.-X. Lei, Y.-W. Zheng, W.-J. Niu, Y.-Z. Wu, B.-N. Gu, M.-J. Liu, F. Ran, Y.-L. Chueh, Design of atomic cobalt selenide-doped sulfurized polyacrylonitrile cathode with enhanced electrochemical kinetics for high performance lithium-SPAN batteries, *Chem. Eng. J.* **2023,** 471, 144581.

[3] L. Wang, H. Shi, Y. Xie, Z.-S. Wu, Boosting solid–solid conversion kinetics of sulfurized polyacrylonitrile via MoS_2_ doping for high-rate and long-life Li-S batteries, *Carbon Neutr.* **2023,** 2, 262.

[4] B. He, Z. Rao, Z. Cheng, D. Liu, D. He, J. Chen, Z. Miao, L. Yuan, Z. Li, Y. Huang, Rationally Design a Sulfur Cathode with Solid-Phase Conversion Mechanism for High Cycle-Stable Li–S Batteries, *Adv. Energy Mater.* **2021,** 11, 2003690.

[5] Y. Li, S. Zhang, H. Liu, Y. Zhang, X. Zhang, Effects of Fiber Diameter on Sulfur Loading and Lithium–Sulfur Battery Performance of Semicarbonized and Sulfurized Polyacrylonitrile Cathode Materials, *ACS Appl. Energy Mater.* **2023,** 6, 8511.

[6] S. Ma, Z. Zhang, Y. Wang, Z. Yu, C. Cui, M. He, H. Huo, G. Yin, P. Zuo, Iodine-doped sulfurized polyacrylonitrile with enhanced electrochemical performance for lithium sulfur batteries in carbonate electrolyte, *Chem. Eng. J.* **2021,** 418, 129410.

[7] H. Liu, Y. Zhang, Y. Li, N. Han, H. Liu, X. Zhang, Solid-State Transformations of Active Materials in the Pores of Sulfurized-Polyacrylonitrile Fiber Membranes via Nucleophilic Reactions for High-Loading and Free-Standing Lithium–Sulfur Battery Cathodes, *Adv. Fiber Mater.* **2024,** 6, 772.

[8] X. Yuan, B. Zhu, J. Feng, C. Wang, X. Cai, R. Qin, Feasible Catalytic-Insoluble Strategy Enabled by Sulfurized Polyacrylonitrile with In Situ Built Electrocatalysts for Ultrastable Lithium–Sulfur Batteries, *ACS Appl. Mater. Interfaces* **2021,** 13, 50936.

[9] R. He, H. Liu, Q. Gao, D. Cai, K. Xiao, Y. Zhang, H. Nie, Y. Liu, L. Kang, Z. Yang, Synthesis and redox mechanism investigation of high sulfur content sulfurized polyacrylonitrile for lithium‑sulfur batteries, *J. Colloid Interface Sci.* **2026,** 703, 139067.

[10] Q. Wu, W. Zhang, S. Li, W. Zhong, H. Zhu, Z. Zeng, C. Yu, S. Cheng, J. Xie, Electrospun Sulfurized Polyacrylonitrile Nanofibers for Long-Term Cycling Stability and High-Rate Lithium–Sulfur Batteries, *ACS Appl. Energy Mater.* **2022,** 5, 5212.
